# Subject-Specific Cardiovascular Signatures Revealed by Arterial Pulse Signals in Patients with Heart Diseases—Part II: Special Cases

**DOI:** 10.3390/diagnostics16213451

**Published:** 2026-10-22

**Authors:** Mamun Hasan, Md Mahfuzur Rahman, Jennifer F. May, John M. Herre, Leryn J. Reynolds, Zhili Hao

**Affiliations:** 1 Department of Mechanical and Aerospace Engineering, Old Dominion University, Norfolk, VA 23529, USA; mhasa004@odu.edu (M.H.); mrahm009@odu.edu (M.M.R.); 2 Sentara Health Research Center, Virginia Beach, VA 23464, USA; jlfreema@sentara.com; 3 Department of Medicine, Division of Cardiology, Macon and Joan Brock Virginia Health Sciences at Old Dominion University, Norfolk, VA 23501, USA; jmherre@sentara.com (J.M.H.); lreynold@odu.edu (L.J.R.); 4 Advanced Heart Failure and Heart Transplantation, Sentara Norfolk General Hospital, Norfolk, VA 23507, USA; 5 School of Exercise Science, Old Dominion University, Norfolk, VA 23529, USA

**Keywords:** arterial pulse signal, arterial pulse waveform, harmonic amplitude, time-frequency analysis, single-degree-of-freedom (SDOF), heart rate variability, respiration

## Abstract

**Aim**: This study evaluates whether cardiovascular (CV) parameters derived from noninvasive arterial pulse signals can reveal subject-specific characteristics in patients with atypical CV dynamics. **Methods**: Seven special-case patients—including a heart transplant (HTx) recipient and individuals with bradycardia (low heart rate, HR), low dicrotic notch (DN), and intermittent arterial pulse waveform (APW) variations—were analyzed across three visits over the cardiac rehabilitation (CR) period. Radial artery (RA) and carotid artery (CA) signals, acquired using micro-fabricated tactile sensors, were processed using a single-degree-of-freedom time–frequency (SDOF–TF) framework to extract multiple CV parameters. The analysis focused on descriptive characterization of subject-specific CV patterns in individual patients. **Results**: The patients exhibited highly heterogeneous CV profiles. Individuals with low HR exhibited relatively limited post-exercise HR changes. Individuals with low DN morphology exhibited elevated lower-order harmonic amplitudes. Individuals with intermittent APW variations exhibited cyclic changes in APW morphology at rest and during post-exercise recovery. Although CV patterns at rest differed across individuals, post-exercise assessment revealed more distinctive individual-specific differences, including differences between individuals within the same phenotype-based case subset. Across all patients and visits, RA measurements provided more stable and interpretable subject-specific CV patterns, whereas CA measurements were more susceptible to motion artifacts (MA) and sensor noise. For individuals with intermittent APW variations, the CA signals exhibited extremely poor signal quality, limiting their suitability for physiological interpretation. **Conclusions**: The SDOF–TF framework enabled the characterization of subject-specific, multi-parameter CV dynamics in these seven special cases. The findings illustrate the potential value of arterial pulse analysis for the individualized characterization of atypical CV dynamics. Future studies involving larger patient cohorts are needed to further investigate these findings and establish their clinical utility.

## 1. Introduction

Following Part I [1], which examined cardiovascular (CV) characteristics in 18 patients with heterogeneous heart diseases, this study focuses on a subset of seven cases analyzed separately from the predominant cohort patterns because of their atypical CV and arterial pulse waveform (APW) profiles. These subjects were identified based on distinctive individual characteristics involving heart rate (HR), the location of the dicrotic notch (DN), and APW morphology. They exhibit patterns that differ from those observed in many of the other patients, including unusually low at-rest HR, atypical DN location, intermittent or irregular APW variability, and distinct clinical conditions such as heart transplantation (HTx). Collectively, these features warrant separate analysis to further characterize subject-specific CV profiles.

Part I [1] aimed to characterize multiple CV parameters and subject-specific CV patterns in a general cardiac rehabilitation (CR) cohort of 18 patients with heterogeneous heart diseases using the single-degree-of-freedom time–frequency (SDOF–TF) framework applied to measured arterial pulse signals. Arterial pulse signals were acquired using microfabricated tactile sensors. Signal-quality screening was performed to identify usable signal segments for the reliable extraction of multiple CV parameters. All radial artery (RA) measurements across the patients and visits were successfully acquired with acceptable signal quality, providing a reliable measurement basis for CV characterization. In contrast, carotid artery (CA) measurements were more susceptible to motion artifacts (MA) and sensor noise, making them less reliable for CV parameter extraction and physiological interpretation. The analysis characterized subject-specific CV patterns at rest and during post-exercise recovery, including variations in time-based CV indices and APW morphology across measurement sites and visits. This work established a descriptive basis for CV characterization in patients with heart disease without attempting to establish population-level differences or statistical associations. Building on Part I, the present study extends this characterization to seven selected atypical cases that warrant closer individual-level examination.

Seven such cases are examined from the same cohort: one patient included due to the distinct clinical context of heart transplantation (HTx) [2,3,4,5,6,7], rendering this subject clinically distinct despite not exhibiting markedly abnormal APW morphology; two patients with low at-rest HR (i.e., bradycardia) [8,9]; two patients with lower relative DN locations within the waveform [10,11]; and two patients exhibiting intermittent APW variations across consecutive pulse cycles [12,13,14]. Given these distinctive characteristics, the cases are analyzed separately, with particular emphasis on comparisons between RA and CA measurements to assess whether the two measurement sites provide complementary information about APW morphology and subject-specific CV patterns. These comparisons are interpreted in light of differences in signal quality, as CA measurements are more susceptible to MA and sensor noise than RA measurements.

The objective of this study is to apply the same methodology adopted in Part I to these cases. Whereas Part I examined CV characteristics across the 18-patient cohort, Part II extends the analysis to seven selected atypical cases whose CV characteristics or APW profiles depart from the predominant cohort patterns. The specific aim is to assess whether the adopted methodology can capture subject-specific CV signal characteristics in these cases, including atypical waveform morphology, intermittent APW variations, longitudinal variability, and site-dependent differences between RA and CA measurements. This case-based analysis therefore extends the characterization performed in Part I by examining whether the SDOF–TF framework applied to arterial pulse signals acquired using tactile sensors can characterize multiple CV parameters and capture subject-specific CV patterns in atypical cases. The findings are intended to provide preliminary evidence of feasibility in characterizing atypical CV patterns, rather than to establish diagnostic or prognostic performance.

## 2. Materials and Methods

### 2.1. Study Population

The seven patients analyzed in Part II were selected from the 25 patients recruited to the outpatient cardiac rehabilitation (CR) study described in Part I. Patient recruitment and measurement procedures were approved by the Eastern Virginia Medical School, Virginia Health Sciences IRB at Old Dominion University (IRB Approval #19-04-FB-0100), and written informed consent was obtained from all participants. The selected seven cases included:

1. CR-008: One heart transplantation (HTx) patient, included because of the distinct clinical context of HTx, despite not exhibiting markedly abnormal APW morphology. This patient was previously described in a case study [15].
2. CR-002 and CR-011: Two patients with relatively low dicrotic notch (DN) locations within the APW.
3. CR-013 and CR-035: Two patients with low at-rest heart rate (HR), consistent with bradycardia.
4. CR-019 and CR-020: Two patients exhibiting intermittent APW variations across consecutive pulse cycles.

Arterial pulse signals were acquired simultaneously at the RA and CA using two identical micro-fabricated tactile sensors, with a polydimethylsiloxane (PDMS) layer placed between the sensor and skin for all CA measurements to improve conformity with the artery. Patient measurements were obtained at rest and at 5, 10, 20, 30, and 60 min post-exercise during Visits 1, 2, and 3, scheduled in the first, second, and fourth weeks of the CR program, respectively. CR008 did not participate at visit 3. As in Part I, the 60-min post-exercise measurements were excluded from analysis because patient activity during the preceding interval was uncontrolled and the signals exhibited substantially larger fluctuations than those at earlier time points.

Previous studies have shown that post-exercise HRV and autonomic modulation may remain altered during the first 5–10 min and, depending on exercise intensity, may not return to baseline within 30 min [16,17,18]. Accordingly, the 5 and 10 min time points were used to characterize relatively early recovery responses, while the 30 min time point provided a later assessment of the persistence or attenuation of subject-specific CV changes. These sampling windows were selected to support descriptive characterization of recovery dynamics and were not intended to represent validated diagnostic time points or thresholds for impaired CV function.

The same CR008 patient and healthy reference described in [15] were included in the present analysis. Except for the exercise protocol, measurement location, and one post-exercise time point, the measurement protocol and tactile sensors used for the healthy reference were identical to those used for the patients. The healthy reference protocol was approved by the Old Dominion University (ODU) Institutional Review Board (IRB #IRB24-166), and written informed consent was obtained. The healthy reference was measured during a single visit at ODU, with measurements obtained at rest and at 5, 10, and 25 min post-exercise. The 25 min post-exercise measurements of the healthy reference were used for comparison with the patients’ 30 min post-exercise measurements. Furthermore, the maximum HR during exercise of the healthy reference was not recorded, and thus the maximum HR from the 1 min post-exercise measurement was used to represent the maximum HR during exercise [1]. All signals were processed using the same SDOF–TF framework to extract the CV parameters defined in Part I. Table 1 summarizes the baseline demographic and clinical characteristics of the seven selected patients and the healthy reference; all characteristics were recorded before Visit 1.

### 2.2. Exercise Protocol and Intensity Assessment

The exercise protocol and intensity assessment were identical to those described in Part I, Section 2.4 [1]. Briefly, exercise intensity was characterized using the maximum HR, HR_max_, recorded by the ScottCare VersaCare wireless telemetry system during exercise. A medical instrument (Mindray Passport 12) was used to record at-rest HR, HR_medical_, for comparison with the tactile sensor measurements.

The absolute maximum HR difference between HR_max_ and the at-rest HR measured using the RA tactile sensor was ΔHR_max_ = HR_max_ − HR_sensor_. The normalized maximum HR difference, ΔHR_max_/HR = ΔHR_max_/HR_sensor_, was used as a relative indicator of exercise intensity. The Borg Rating of Perceived Exertion (RPE) scale (6–20) was also used in the cardiac rehabilitation (CR) program to assess perceived exertion, with the majority of recorded RPE scores falling within the range of 10–12.

Table 2 summarizes the at-rest HR measurements, HR_max_ during exercise, and corresponding absolute and normalized maximum HR differences for the seven selected patients across the available visits. Patients not taking beta blockers are indicated in bold, as are normalized maximum HR differences exceeding the 68% observed in the healthy reference. The normalized maximum HR difference ranged from 10% to 115% across the recorded patient visits, compared with 68% for the healthy reference, illustrating substantial inter-individual and visit-to-visit variation in the observed HR response during exercise. For example, CR013 exhibited an increase in the normalized maximum HR difference from 34% at Visit 1 to 88% at Visit 3, whereas CR035 showed comparatively smaller differences of 11%, 28%, and 15% across the three visits. CR020 exhibited the highest normalized HR difference, 115%, at Visit 3.

These comparisons are descriptive and should be interpreted in the context of differences in baseline HR, CV conditions, medication use, and exercise conditions. Exercise intensity was not standardized across patients, and the healthy reference completed a separate exercise protocol, as described in Part I. Notably, at Visit 3, only CR013, who was not taking a beta blocker, and CR020, who was taking a beta blocker, exhibited normalized maximum HR differences exceeding the 68% observed in the healthy reference. Thus, normalized maximum HR differences exceeding this reference value were observed in patients both with and without beta-blocker use. However, given the small number of cases and the heterogeneity of clinical characteristics and exercise conditions, these observations do not establish differences in exercise capacity or the independent effect of beta-blocker use on exercise-related HR responses.

### 2.3. Analysis Strategy

The same SDOF–TF framework used in Part I was applied to both RA and CA signals in these seven cases. The tissue-contact-sensor (TCS) stack between the sensor and the artery was modeled as an SDOF system to account for its dynamic behavior during pulse measurement. Time-frequency (TF) analysis was then applied to the measured pulse signals to account for their nonstationary nature and extract the instantaneous parameters of each harmonic [15]. The same signal-quality screening procedure described in Part I, which evaluates the contributions of MA and sensor noise to the measured pulse signals, was applied to the signals from all seven patients. The extracted CV parameters were identical to those in Part I and are listed below:

- Heart rate (HR) averaged from the first three harmonics
- Heart rate variability (HRV) averaged from the first three harmonics, with HRV of each harmonic being calculated as root mean squared error (RMSE) of instantaneous HR relative to the average HR over the analyzed pulse signal period
- HRV induced by physiological factors (PF), other than respiration, (HRV_PF_) averaged from the first three harmonics
- Respiration rate (RR) averaged from the first three harmonics
- Respiration modulation (RM) of each of the first three harmonics
- Normalized APW (relative to the pulse amplitude) with constant HR, where HRV is removed to avoid APW variation across pulse cycles
- Normalized amplitude (*A_i_*) of the *i*th harmonic (relative to the amplitude of the first harmonic) of the 2nd~7th harmonics (*i* = 2~7)
- Normalized amplitude change (Δ*A_i_*) of the *i*th harmonic 5 min and 10 min post-exercise (relative to the corresponding at-rest value) of the 2nd~7th harmonics (*i* = 2~7)

Signal quality was assessed using the procedure described in Part I. The TCS stack between the sensor and the artery introduces mechanical interactions among the artery, surrounding tissue, and sensor structure. MA can cause baseline drift (BD) and time-varying system parameters (TVSP) within the TCS stack. BD, which reflects low-frequency motion, can be reduced through low-pass filtering, whereas TVSP-induced distortion affects each individual harmonic in a measured pulse signal and is not readily removed by conventional frequency filtering. The SDOF–TF framework was therefore used to recover pulse-induced signals from measurements affected by TVSP-induced distortion and sensor noise, followed by qualitative signal-quality assessment.

RA signals generally provided more usable segments with low MA for CV parameter extraction, whereas CA signals were more susceptible to high MA and TVSP-induced distortion. Time-based indices, HR, HRV, RR, and RM, are relatively robust to TCS stack variations; therefore, RA-derived time-based indices were used for quantitative comparison across subjects and across visits. CA signal quality varied across cases and was particularly poor in the two cases exhibiting intermittent APW variations (CR019 and CR020), as described in Section 3. The CA segments with the lowest MA were selected for parameter extraction. Nevertheless, CA time-based indices were considered insufficiently reliable for physiological interpretation and are presented alongside their RA counterparts for descriptive comparison only.

In contrast, normalized APW morphology, including the upstroke slope and DN location, and harmonic amplitude distributions were considered for qualitative within-subject comparisons across visits, although they were less reliable at the CA than at the RA. Because these waveform characteristics depend on TCS system parameters that may vary across subjects, they were not used for direct cross-subject comparisons or interpreted as direct indicators of arterial stiffness.

Given the relatively high reliability of RA-derived HR measurements, small between-visit changes of RA-derived CV parameters may reflect physiological variation, measurement variability, or a combination of both. Moderate-to-large changes would be expected to reflect physiological variation to a greater extent, with a relatively smaller contribution from measurement variability.

The analysis followed the strategy described in Part I and comprised three complementary components:

1. Subject-specific characterization: The extracted CV parameters were examined to identify distinctive CV patterns and atypical features in each of the seven selected cases.
2. Longitudinal comparison: Changes across repeated visits were evaluated descriptively, with attention to subject-specific trends in the extracted parameters and waveform characteristics.
3. Cross-case and cross-site comparison: CV patterns were compared qualitatively across cases to identify similarities and differences among the selected patients. RA–CA comparisons were conducted descriptively to examine whether distinctive waveform features observed at the CA were also apparent at the RA. These comparisons were not intended to establish quantitative equivalence between RA and CA measurements.

Given the small number of selected cases, their heterogeneous clinical characteristics, and the absence of reference physiological measurements for validation, the analyses were descriptive and were not intended to support population-level statistical inference or clinical diagnosis.

The single healthy reference was used as a device-specific baseline for descriptive comparison with the selected CV patients, rather than as a normative reference representative of the healthy population. The use of the same measurement instrument for the healthy reference and patients minimized potential measurement differences attributable to different instruments. However, comparisons of APW morphology and harmonic amplitude distributions were interpreted cautiously because these characteristics depend on TCS system parameters that may vary across subjects.

ChatGPT-6 (OpenAI) was used to polish author-written manuscript text for grammar, clarity, and readability. All scientific content, data analysis, interpretation, and conclusions were determined by the authors.

## 3. Results

### 3.1. Heart Rate (HR) and Heart Rate Variability (HRV)

**Figure 1:**
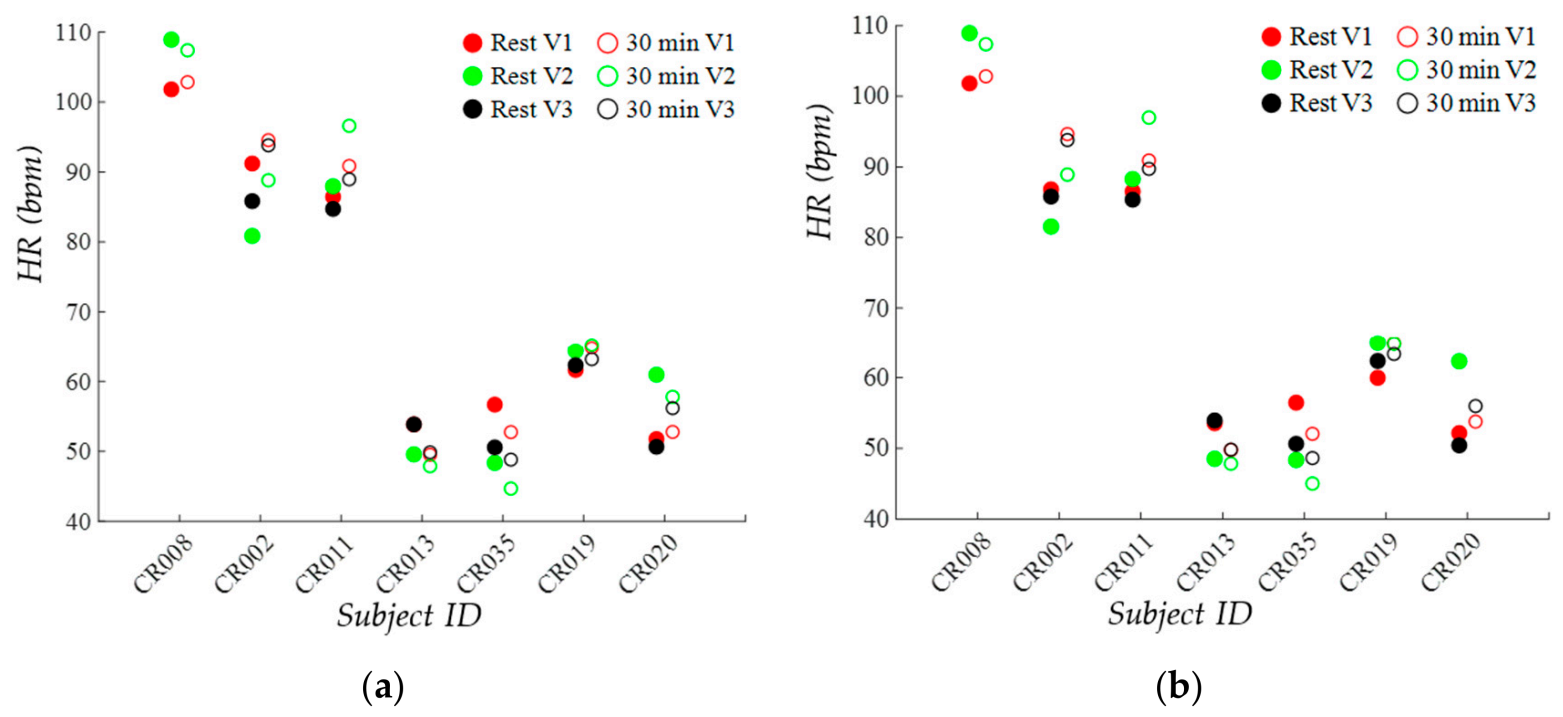
HR at rest and 30 min post-exercise of the subjects: (**a**) RA, (**b**) CA.

As shown in Figure 1, CR008 (HTx) exhibited the highest at-rest HR, at approximately 100 bpm. This finding is consistent with reports of elevated at-rest HR following heart transplantation [1,2,3,4,5,6], which is commonly associated with cardiac denervation and the loss of direct parasympathetic vagal modulation. This characteristic pattern was observed in CR008 throughout the study.

The low-DN case subset (CR002 and CR011) exhibited at-rest HR values within the usual adult at-rest range. In contrast, the low-HR case subset (CR013 and CR035) exhibited bradycardia, with at-rest HR values of 55 bpm and 50 bpm, respectively. CR020 also exhibited an at-rest HR of 50 bpm, although this patient was not included in the low-HR case subset.

Two patterns were observed in the HR measurements. First, at-rest HR remained relatively stable across visits for all subjects, with only modest longitudinal variation. Second, HR at rest and 30 min post-exercise were generally comparable, suggesting that HR had approached its baseline level by this time point. These observations are descriptive, given the variability in exercise protocols and the absence of quantitative measurement-error assessment.

Another notable finding was the similarity between HR estimates derived from RA and CA measurements, both at rest and 30 min post-exercise. This cross-site agreement suggests that both measurement sites captured similar overall cardiac rate patterns in the selected cases. Overall, no large longitudinal changes in HR were observed across visits in any of the seven cases. These changes were assessed qualitatively because measurement variability was not quantitatively characterized; therefore, the relative contributions of physiological variation and measurement variability could not be determined quantitatively.

HR also appeared less sensitive to CA signal-quality limitations than time-based indices such as HRV, RR, and RM. These latter indices are more susceptible to TVSP-induced temporal variations that affect the instantaneous characteristics of the measured pulse signal. In contrast, HR is an aggregate measure derived from the instantaneous frequencies of the first three harmonics and may therefore be less affected by these distortions. Nevertheless, the lower reliability of CA signals should be considered when interpreting CA-derived parameters, particularly HRV, RR, and RM.

**Figure 2:**
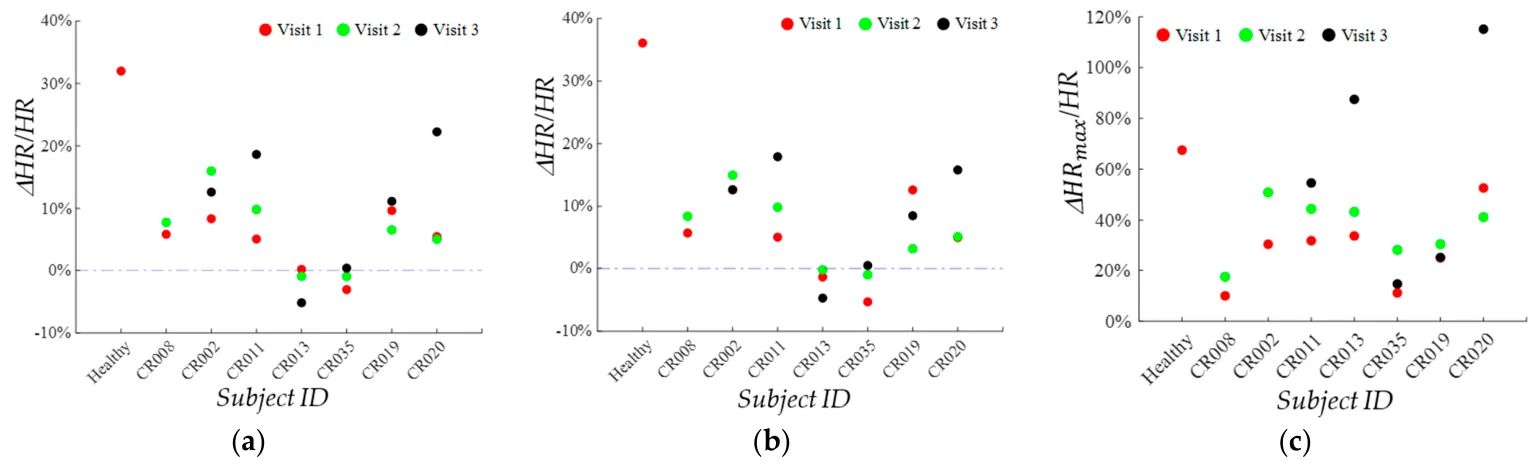
The highest ΔHR/HR of the subjects during post-exercise recovery (occurring at 5 min post-exercise) at (**a**) RA and (**b**) CA. (**c**) The ΔHR_max_/HR reached during exercise, used as a relative indicator of exercise intensity.

As shown in Figure 2a,b, the immediate CV response to exercise was quantified as the relative change in HR at 5 min post-exercise compared to the at-rest baseline (ΔHR/HR). A key observation was a markedly reduced HR response in the special-case cohort, with maximal ΔHR/HR values substantially lower than those observed in the healthy reference. Notably, CR013 and CR035 exhibited negative ΔHR/HR values, indicating that their HR at 5 min post-exercise fell below baseline levels.

For the remaining subjects, ΔHR/HR remained positive but modest, with values below the increase observed in the healthy reference. CR011 and CR020 exhibited relatively large longitudinal changes across visits, whereas the other subjects showed comparatively small variations over time.

Similar post-exercise patterns of ΔHR/HR were observed at both RA and CA measurement sites, suggesting that the ΔHR/HR metric captured broadly consistent HR responses across the two sites. However, this cross-site agreement does not establish that the observed changes arose exclusively from physiological variation. Small differences in ΔHR/HR between the two arterial sites were also observed and may have been associated with the greater susceptibility of CA measurements to MA at 5 min post-exercise. Because measurement variability was not quantitatively assessed, these interpretations remain qualitative.

Figure 2c shows the normalized maximum HR increase, ΔHR_max_/HR, during exercise for each subject across visits, used as a descriptive indicator of relative exercise intensity. Overall, the post-exercise ΔHR/HR at 5 min followed a pattern broadly consistent with the corresponding ΔHR_max_/HR during exercise for each subject across visits, with the exception of CR013. For CR013, ΔHR_max_/HR increased markedly from Visit 1 to Visit 3, whereas the post-exercise ΔHR/HR at 5 min remained close to or below zero. Notably, CR013 was taking a beta blocker. In contrast, CR008, the other subject taking a beta blocker, exhibited both a relatively low exercise intensity and a low ΔHR/HR at 5 min post-exercise. These differing patterns indicate that the observed HR responses cannot be attributed to beta-blocker use alone and may also reflect differences in individual physiology and exercise response. Given the limited number of cases, these observations are descriptive and do not establish a causal relationship.

**Figure 3:**
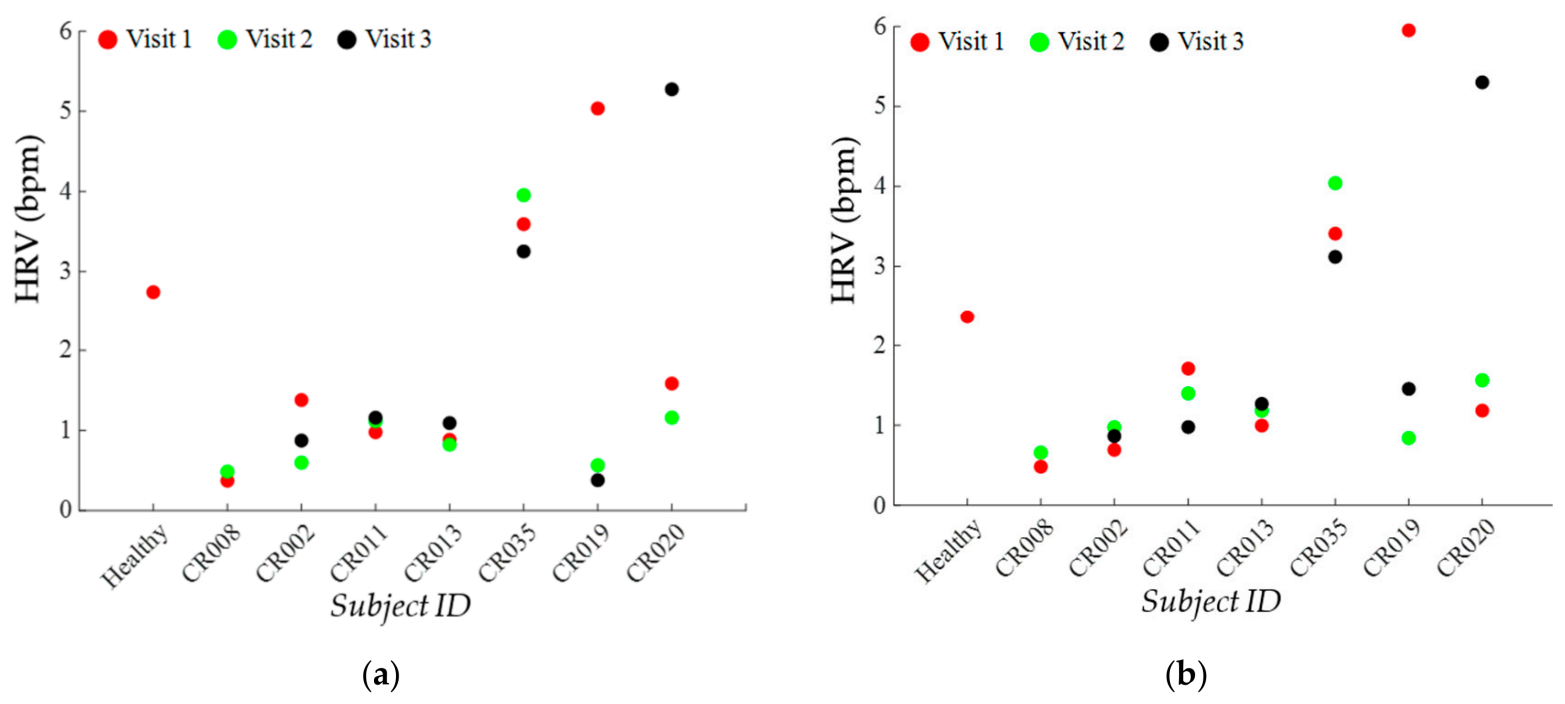
HRV at rest of the subjects: (**a**) RA, (**b**) CA.

As illustrated in Figure 3, CR008 (HTx) exhibited markedly reduced at-rest HRV, consistent with the known effects of heart transplantation on CV autonomic regulation. The low-DN case subset (CR002 and CR011) also exhibited relatively low at-rest HRV. In contrast, at-rest HRV varied within the low-HR case subset: CR013 exhibited low HRV, whereas CR035 exhibited higher HRV than the healthy reference.

Most subjects maintained longitudinally stable at-rest HRV across the three visits. Exceptions were CR019 and CR020, who displayed substantial variability over time. Notably, CR019 (AF and MI)— the only subject with AF—demonstrated a large swing in at-rest HRV across visits. Although AF is typically associated with elevated at-rest HRV, CR019 showed a high at-rest HRV only during the first visit. Similarly, CR020 (PCI) exhibited pronounced variability, with an exceptionally high at-rest HRV observed at the final visit.

At-rest HRV remained relatively stable across the three visits for most subjects. The main exceptions were the case subset with intermittent APW variations (CR019 and CR020), both of whom exhibited substantial longitudinal variability. CR019, the only subject with atrial fibrillation (AF), also had a history of myocardial infarction (MI) and showed marked visit-to-visit changes in at-rest HRV. Although AF is often associated with elevated HRV estimates because of irregular ventricular intervals, CR019 exhibited high at-rest HRV only at Visit 1. Similarly, CR020 (PCI) showed pronounced longitudinal variability, with an exceptionally high at-rest HRV value at the final visit. These findings illustrate substantial within-subject variability in HRV across visits, particularly in these two cases. However, because reference ECG measurements were not available for validating the extracted HRV values, the relative contributions of physiological variation and measurement variability cannot be determined.

For CR019 (AF and MI), intermittent APW variations at rest were observed only at Visit 1, coinciding with markedly elevated HRV. HRV decreased at Visits 2 and 3 as the APW became more stable (see Figure 11). Conversely, CR020 exhibited minimal intermittent APW variations at rest during Visits 1 and 2, followed by pronounced waveform fluctuations at Visit 3 (see Figure 12). This pattern corresponded to relatively low at-rest HRV during the first two visits and a substantial increase at the final visit. The mechanisms underlying these dynamic changes remain uncertain. Potential contributing factors include changes in physiological status, medication adjustments, or differences in exercise-related responses across visits; however, the available data do not establish a specific explanation.

The strong concordance of at-rest HRV values between RA and CA measurements underscores the reliability of the methodology, supporting the conclusion that the large longitudinal swings observed in CR019 and CR020 represent true physiological changes rather than measurement variability. As noted in Part I, the mild differences in at-rest HRV between the two arterial sites likely arise because MA is more pronounced at the CA site, and metrics related to fine features in time-domain—such as HRV, RR, and RM—are more sensitive to such artifacts than APW and HR.

The overall agreement between at-rest HRV values derived from RA and CA measurements suggests that both sites captured similar longitudinal patterns. This cross-site agreement lends support to the interpretation of the substantial HRV changes observed in CR019 and CR020, although it does not establish that these changes were exclusively physiological rather than partly attributable to measurement variability. As noted in Part I, the modest differences in at-rest HRV between the two arterial sites may be associated with the greater susceptibility of CA measurements to MA. Time-based indices that depend on instantaneous pulse dynamics, such as HRV, RR, and RM, are more sensitive to MA and sensor noise than aggregate HR and APW morphology.

Notably, the CA measurements for CR019 and CR020 were affected by substantial MA and sensor noise (see Supplementary Materials). Nevertheless, the extracted at-rest HR and HRV values showed overall agreement between the RA and CA measurements. This agreement provides qualitative evidence that the SDOF–TF method can recover consistent CV indices from measurements affected by substantial MA and sensor noise. However, because measurement error was not quantitatively assessed, the extent to which these artifacts were removed cannot be determined precisely. Therefore, CA-derived HRV values should be interpreted cautiously, particularly when assessing longitudinal changes.

**Figure 4:**
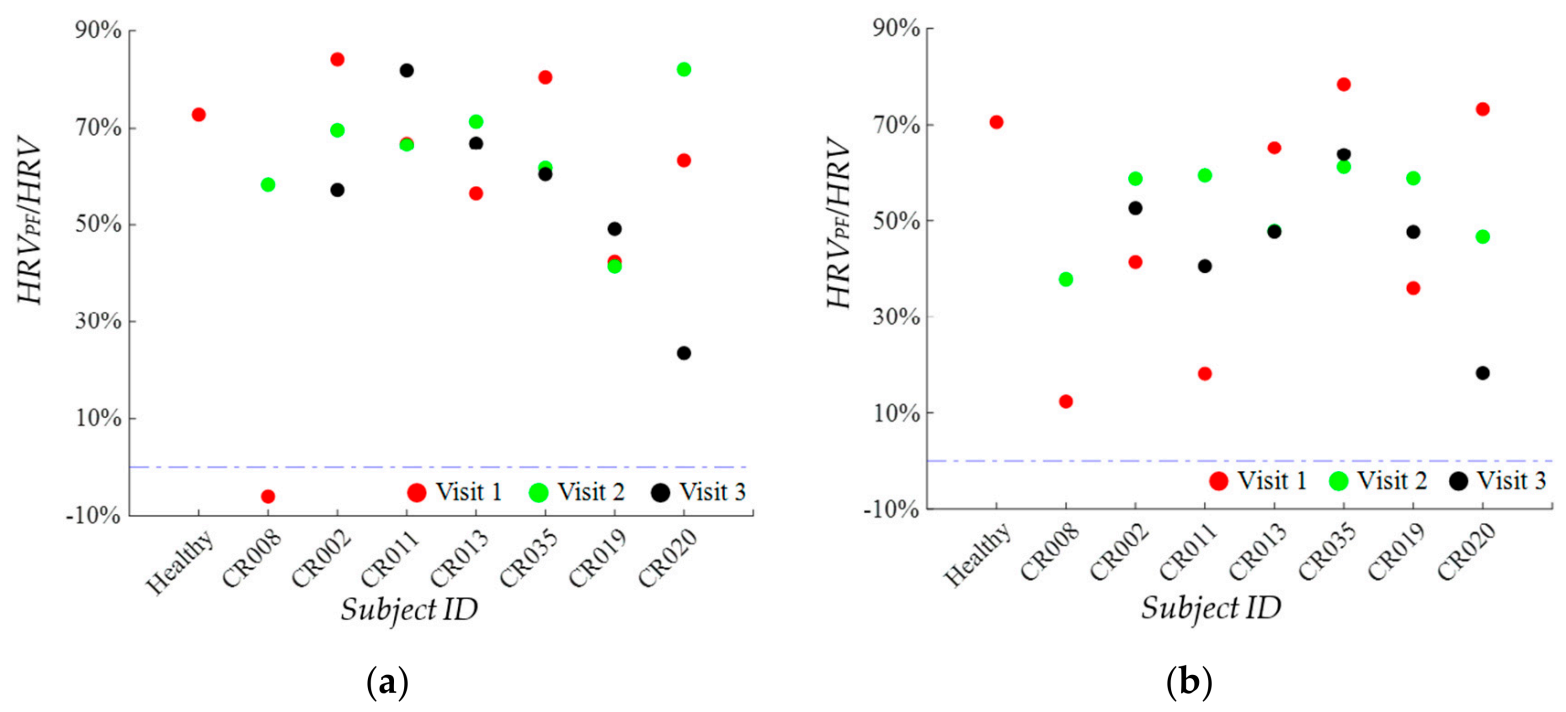
The contribution of PF-induced HRV, HRV_PF_/HRV at rest of the subjects (**a**) RA, (**b**) CA.

The contribution of non-respiratory PF to total HRV, expressed as HRV_PF_/HRV, is illustrated in Figure 4. This ratio represents the relative contribution of the HRV component attributed to non-respiratory factors within the SDOF–TF framework. Such factors may include sympathetic modulation, blood pressure fluctuations, and intrinsic cardiac rhythm variability; however, the ratio does not independently identify or quantify the contribution of any specific mechanism.

Here, we focus on the results derived from RA measurements. For CR008 (HTx), HRV_PF_/HRV increased markedly from Visit 1 to Visit 2. The negative value observed at Visit 1 is attributable to MA affecting the instantaneous frequency extracted from the measured pulse signal and should be interpreted as a very low value rather than a physiologically meaningful negative contribution. The low-DN and low-HR case subsets exhibited HRV_PF_/HRV values at the final visit that were relatively comparable to those of the healthy reference. In contrast, CR019 and CR020 showed markedly lower values than the healthy reference at the final visit.

CR008 and CR020 exhibited the largest longitudinal variations in HRV_PF_/HRV across visits. However, the physiological significance of these changes remains uncertain. Although the relatively small variation observed in CR019 contrasts with the larger fluctuations in CR008 and CR020, the available data do not establish whether the observed differences primarily reflect physiological changes or measurement variability. Finally, notable inconsistencies between RA- and CA-derived HRV_PF_/HRV values were observed. These differences may be associated with the greater susceptibility of CA measurements to MA and sensor noise, which can affect the instantaneous frequency estimates and the subsequent decomposition of HRV. Therefore, CA-derived HRV_PF_/HRV values should be interpreted cautiously.

### 3.2. Respiratory Parameters: RR and RM

**Figure 5:**
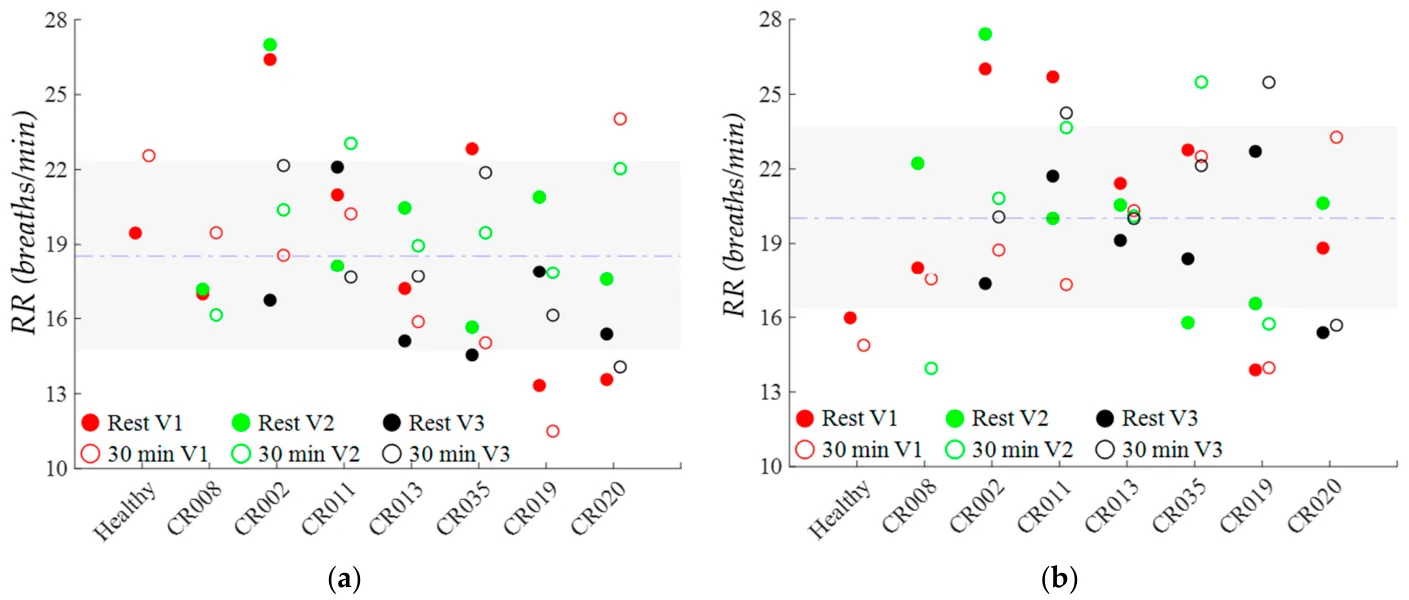
RR at-rest and 30 min post-exercise of the subjects, with the mean ± SD (**a**) RA (**b**) CA. V1, V2, and V3 denote Visits 1, 2, and 3, respectively.

As shown in Figure 5, inter-site differences in RR between at-rest and 30 min post-exercise measurements became more pronounced, possibly reflecting the greater susceptibility of CA measurements to MA. Accordingly, the following analysis focuses solely on RA measurements. Because the absolute magnitude of RR is relatively small, even modest absolute differences across visits may represent proportionally large changes and may be more susceptible to measurement variability. The results from Visit 3 are emphasized below, except for CR008, for which Visit 3 data were unavailable and Visit 2 data were therefore used.

At rest, CR008 (HTx) exhibited RR values lower than those of the healthy reference, with only minimal change at 30 min post-exercise. The low-DN case subset showed divergent responses: CR002 exhibited a low at-rest RR followed by a marked increase at 30 min post-exercise, whereas CR011 exhibited an elevated at-rest RR followed by a substantial reduction at 30 min post-exercise. The low-HR case subset exhibited consistently low at-rest RR values, followed by pronounced increases at 30 min post-exercise, with CR035 showing a greater post-exercise increase than CR013.

CR019 exhibited an at-rest RR comparable to that of the healthy reference, whereas CR020 exhibited a substantially lower at-rest RR. However, both subjects showed moderate reductions in RR at 30 min post-exercise relative to their respective at-rest values. Notably, the combination of low at-rest RR and low at-rest HR in CR020 is consistent with the pattern observed in the low-HR case subset.

**Figure 6:**
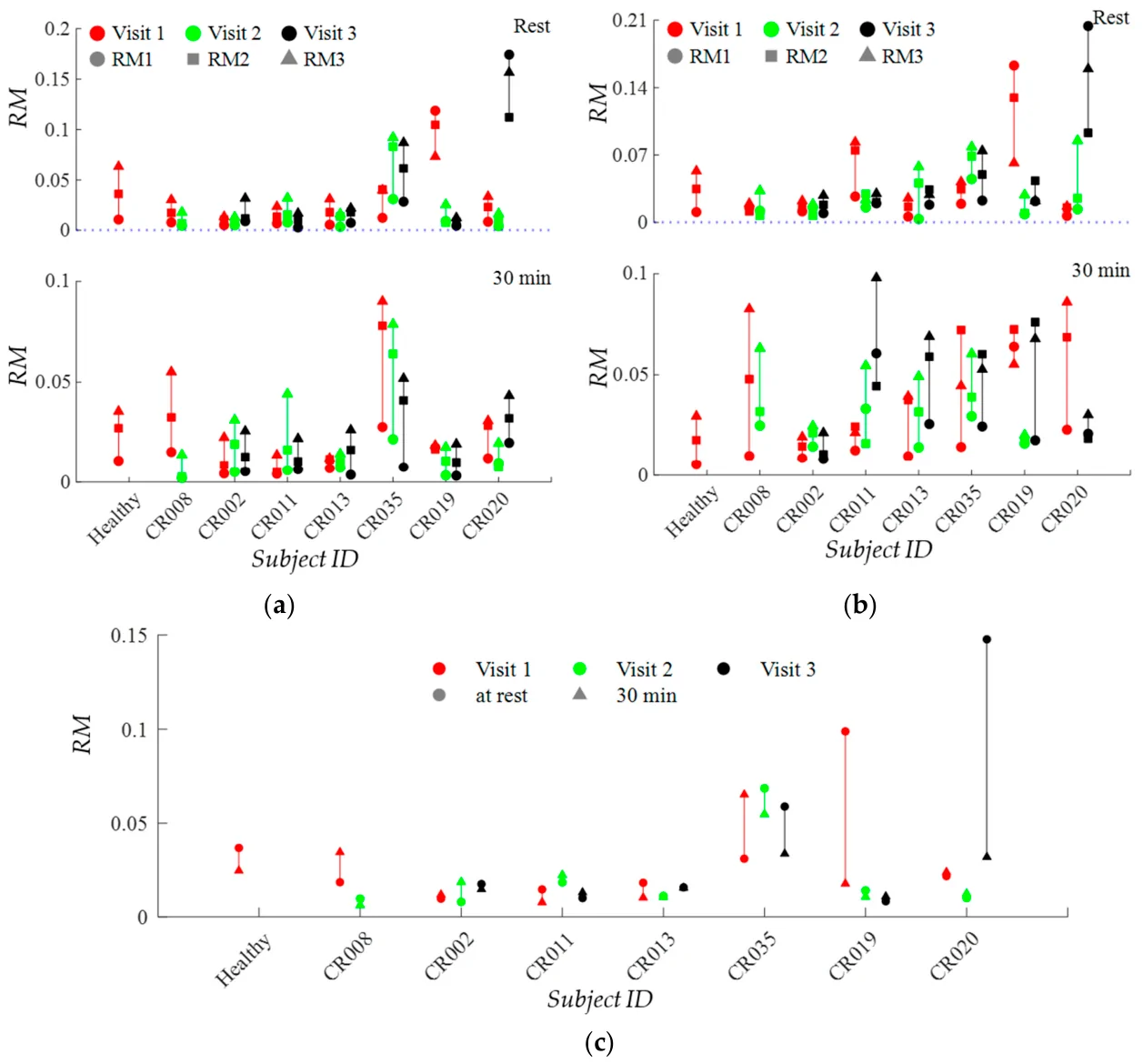
The RM of the subjects: (**a**) at-rest and 30 min post-exercise RM of each harmonic at the RA (**b**) at-rest and 30 min post-exercise RM of each harmonic at the CA; (**c**) averaged RM at-rest and 30 min post-exercise at the RA.

As shown in Figure 6a,b, similar to RR, the greater susceptibility of CA measurements to MA reduces their reliability relative to RA measurements. Therefore, the following analysis focuses exclusively on RM derived from RA measurements. The healthy reference exhibited RM values that increased with harmonic order, with relatively consistent increments between consecutive harmonics.

Compared with the healthy reference, CR008 (HTx) exhibited reduced at-rest RM, consistent with the known effects of heart transplantation on autonomic regulation. The low-DN case subset also exhibited reduced RM. Within the low-HR case subset, CR013 showed low at-rest RM, whereas CR035 exhibited at-rest RM comparable to that of the healthy reference during the last two visits. Notably, CR035 had a CRT-D. Overall, for these five subjects, RM generally increased with harmonic order at-rest and 30 min post-exercise, although the relatively consistent increments between consecutive harmonics observed in the healthy reference were not consistently preserved.

CR019 (AF and MI) exhibited elevated at-rest RM during Visit 1, followed by reduced at-rest RM during Visits 2 and 3. In contrast, CR020 (PCI, 76–80 years) exhibited reduced at-rest RM during the first two visits, followed by a substantial increase during Visit 3. For both subjects, the reduced at-rest RM profiles largely preserved the increasing trend with harmonic order, whereas the elevated at-rest RM profiles showed a less orderly harmonic progression. Nevertheless, at 30 min post-exercise, RM regained an increasing trend with harmonic order in both subjects.

To more clearly illustrate the differences between at-rest and 30 min post-exercise conditions, Figure 6c presents RM values averaged across harmonics. Relative to the at-rest condition, the healthy reference exhibited reduced RM at 30 min post-exercise, consistent with previous reports of diminished respiration-related HRV modulation following exercise [9]. CR008 (HTx) exhibited the opposite response during Visit 1. Although the RM change during Visit 2 followed the same directional trend as that of the healthy reference, its magnitude was minimal. The low-DN case subset showed only slight RM changes between at-rest and 30 min post-exercise. Similarly, CR013 in the low-HR case subset exhibited minimal RM change, whereas CR035 showed a substantially larger change. During Visit 3, CR035 exhibited the same directional pattern as the healthy reference. CR019 during Visit 1 and CR020 during Visit 3 also exhibited markedly larger RM changes, both in the same direction as the healthy reference.

### 3.3. Normalized APW and Harmonic Analysis

#### 3.3.1. Subject-Specific Comparison of Individual Normalized Harmonic Amplitudes at the RA and the CA

**Figure 7:**
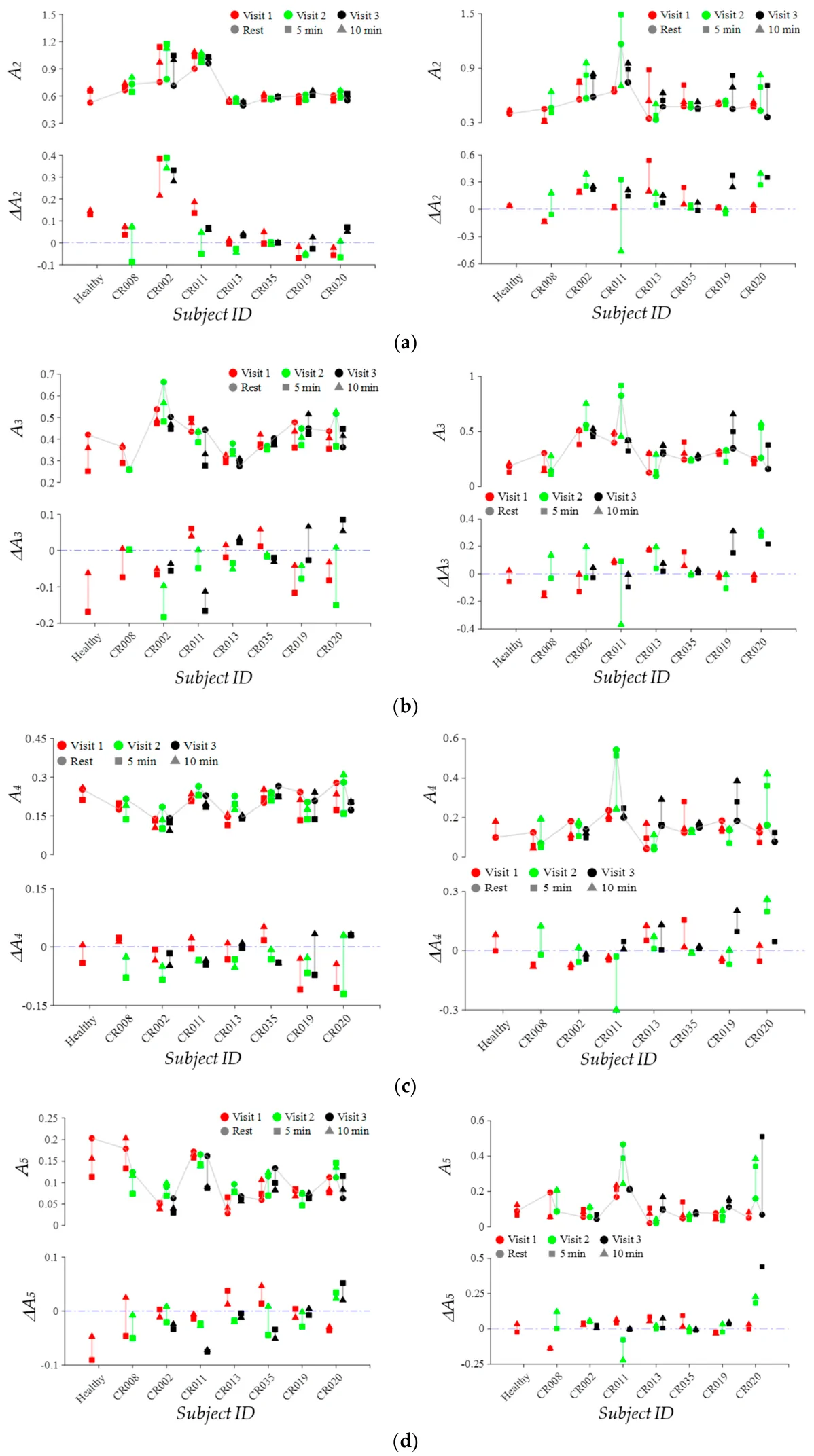

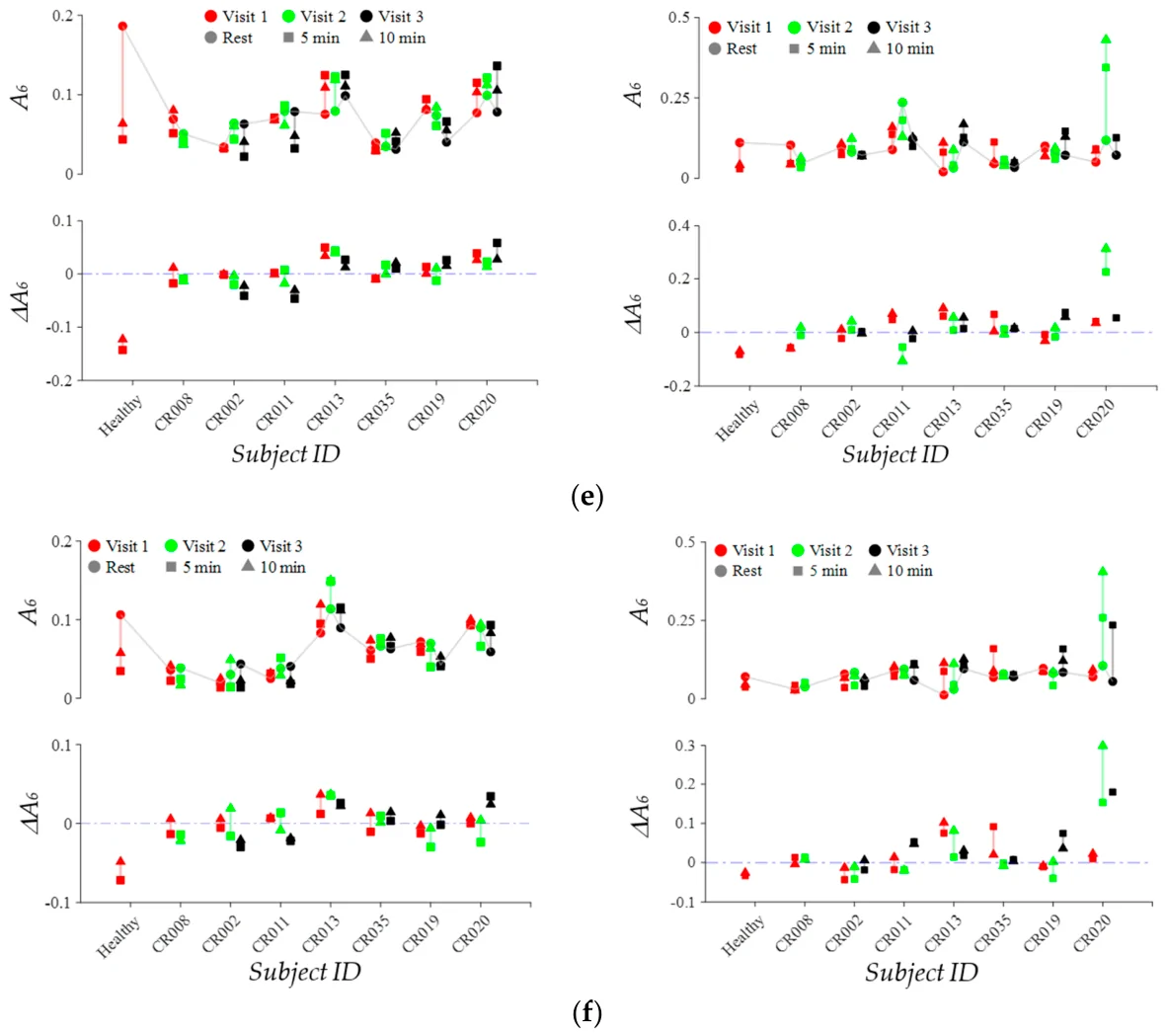
Harmonic amplitudes in response to exercise, at-rest, 5 min and 10 min post-exercise of the subjects across three visits (left figure: RA; right figure: CA) (**a**) 2nd harmonic (**b**) 3rd harmonic (**c**) 4th harmonic (**d**) 5th harmonic (**e**) 6th harmonic (**f**) 7th harmonic.

As shown in Figure 7, the normalized amplitudes of the 2nd through 7th harmonics provide a spectral characterization of the APW at rest and during recovery (5 and 10 min post-exercise). These results illustrate differences in harmonic amplitude distributions across the selected CV cases.

For the healthy reference, differences in the post-exercise harmonic responses were observed between the RA and CA. Nevertheless, both sites showed increases in the 2nd harmonic amplitude and decreases in the 6th and 7th harmonic amplitudes at 5 and 10 min post-exercise. Notably, differences between the patient responses and the healthy reference were more apparent in the RA measurements than in the CA measurements. This observation suggests that RA-derived harmonic amplitudes may provide a clearer basis for descriptive comparisons in this dataset, although the interpretation of harmonic distributions remains subject to differences in TCS system parameters and measurement quality (i.e., the level of MA relative to the pulse-induced signal).

At the RA, all patients exhibited a smaller post-exercise increase in the second harmonic amplitude than the healthy reference, except for CR002 across all visits and CR011 during Visit 1. Changes in the 3rd through 5th harmonics were heterogeneous, whereas changes in the 6th and 7th harmonics were markedly smaller than those of the healthy reference. These harmonic response patterns differed from those observed in the general cases presented in Part I. Furthermore, the harmonic response patterns at both 5 and 10 min post-exercise varied substantially across patients, and the corresponding longitudinal changes also differed markedly among individuals. Notably, CR013 and CR020 experienced exceptionally high exercise intensity at Visit 3, but their harmonic amplitude changes did not show a corresponding increase. This observation suggests that post-exercise harmonic amplitude responses were not directly proportional to exercise intensity in these cases.

In contrast, no consistent harmonic amplitude changes were observed across patients at the CA. This lack of a consistent pattern should be interpreted cautiously because CA-derived harmonic amplitudes are more susceptible to variations in the TCS stack and measurement quality than RA-derived harmonic amplitudes.

#### 3.3.2. Cross-Subject and Longitudinal Comparison of Normalized Harmonic Amplitude Profiles at the RA

**Figure 8:**
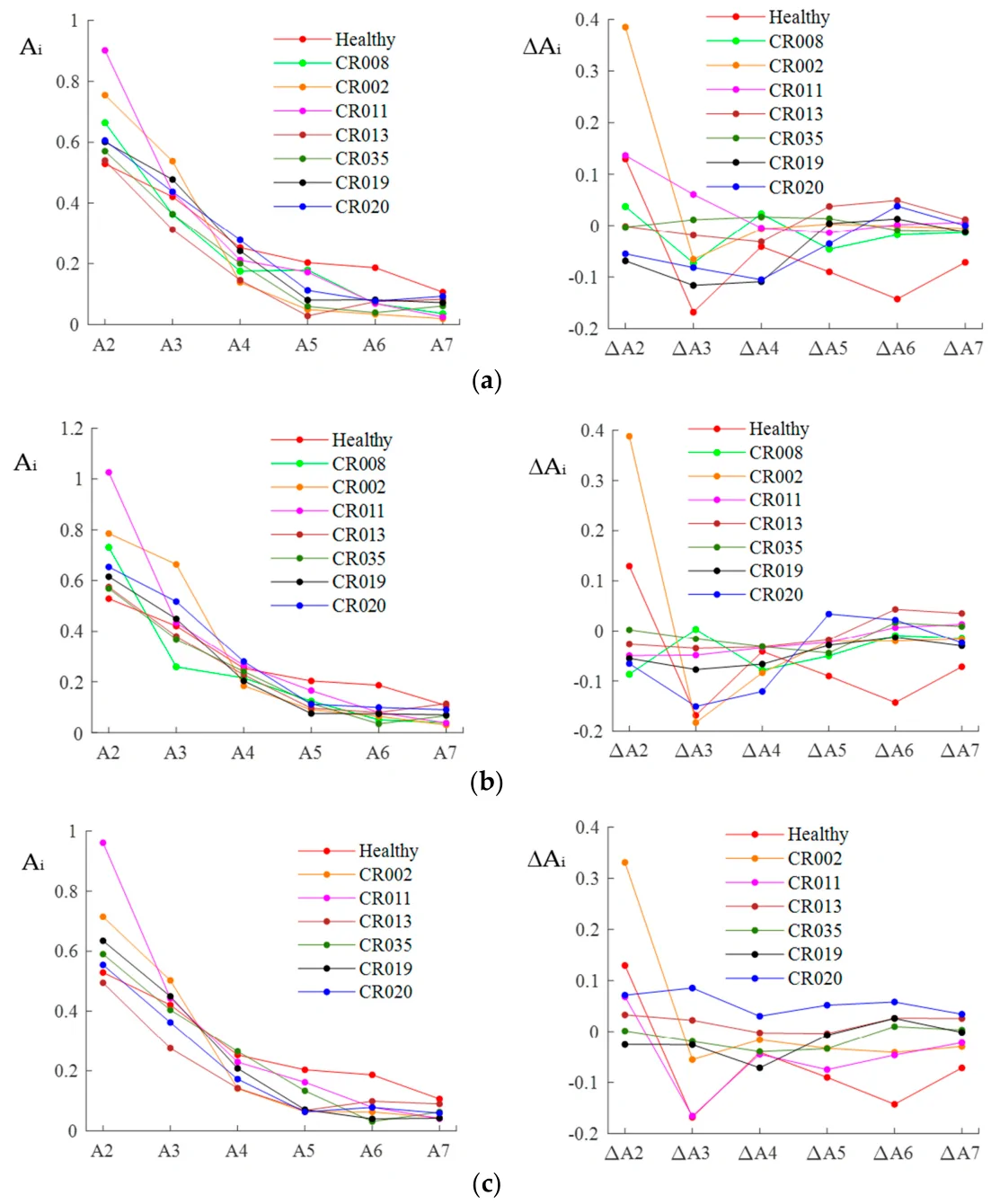
Cross-subject comparison of normalized harmonic amplitude profiling (**a**) Visit 1 (**b**) Visit 2 (**c**) Visit 3 (note that *A_i_* and Δ*A_i_* denote the *i*th normalized harmonic amplitude at-rest and the *i*th normalized harmonic change 5 min post-exercise, relative to at-rest).

As shown in Figure 8, within the low-DN case subset, CR011 exhibited an extremely high second harmonic amplitude, whereas CR002 showed marked increases in both the 2nd and 3rd harmonics. These patterns were consistent with the lower DN locations observed in their APWs. For the remaining patients, the 2nd harmonic amplitude was generally higher than that of the healthy reference across visits. Consistent with the findings in Part I, the 5th through 7th harmonic amplitudes remained lower than those of the healthy reference across visits, whereas the 3rd and 4th harmonics exhibited heterogeneous changes across patients. Overall, at-rest normalized harmonic amplitude profiles showed limited separation among patients, except for the low-DN case subset. The remaining five patients showed no pronounced differences in their harmonic profiles relative to the healthy reference.

In contrast, the 5 min post-exercise responses more clearly differentiated patient-specific harmonic dynamics. CR002 and CR011 exhibited markedly different post-exercise harmonic patterns. CR019 and CR020 also showed distinct post-exercise responses, with noticeable changes across visits. In the low-HR case subset (CR013 and CR035), post-exercise responses were more similar, with only modest longitudinal variation. CR008 (HTx) demonstrated a pronounced difference from the healthy reference in its post-exercise harmonic profile.

**Figure 9:**
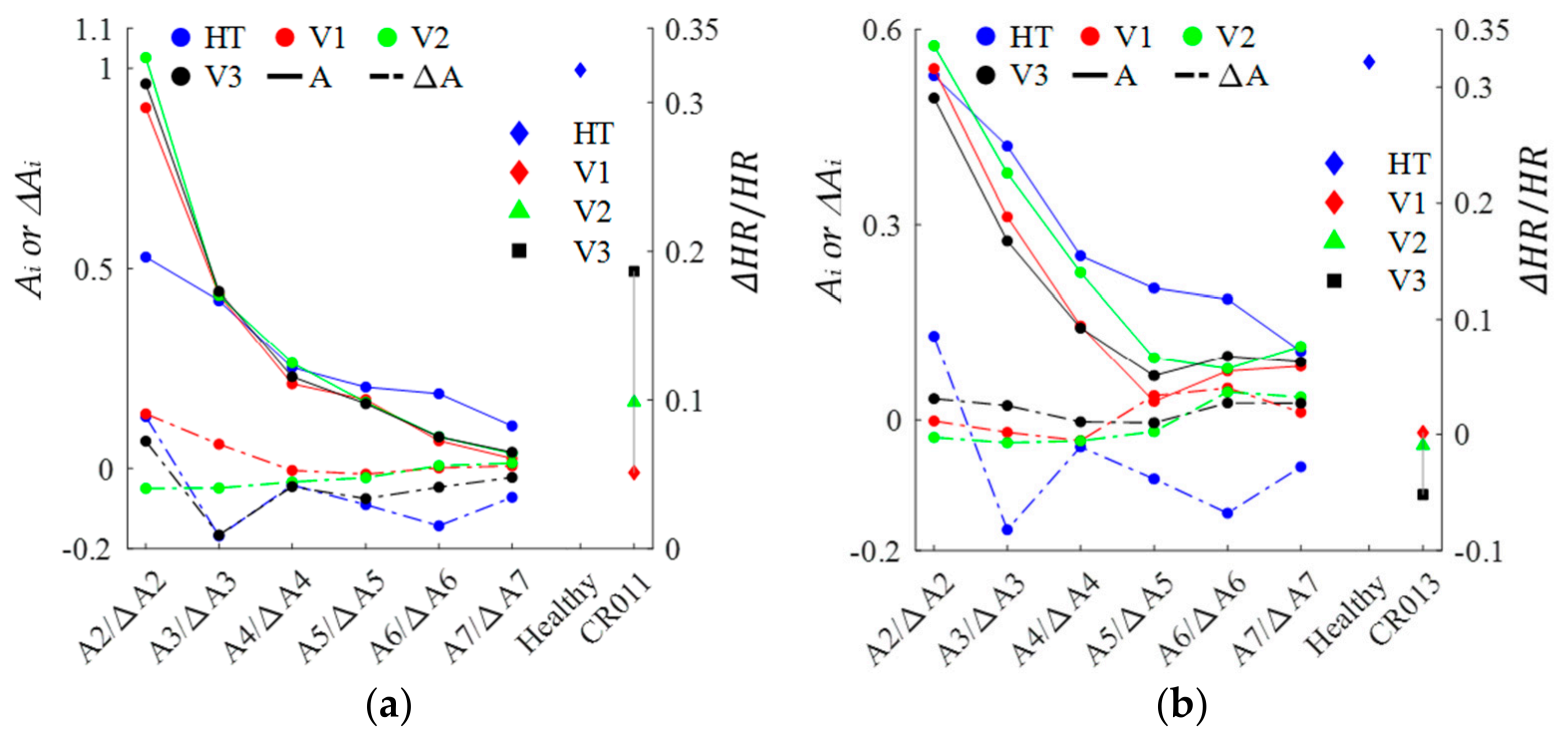
Longitudinal changes of normalized harmonic amplitude profiling (**a**) CR011 (**b**) CR013. *A_i_* and Δ*A_i_* denote the *i*th normalized harmonic amplitude at-rest and the *i*th normalized harmonic change 5 min post-exercise, relative to at-rest and ΔHR/HR is also included. HT denotes the healthy subject; V1, V2, and V3 denote the first, second, and third visits, respectively.

Figure 9 illustrates longitudinal variations in normalized harmonic amplitude profiles at-rest and their responses at 5 min post-exercise for two patients, selected for illustration due to space constraints. CR011 exhibited stable at-rest harmonic amplitude profiles across all visits, with no noticeable longitudinal changes. However, its 5 min post-exercise harmonic response varied substantially between visits. Although ΔHR/HR increased over time, the harmonic amplitude profiles did not track this trend directly. Despite this mismatch, the post-exercise response progressively approached the healthy reference profile.

In contrast, CR013 showed longitudinal variability in both at-rest and 5 min post-exercise harmonic profiles, with persistent differences from the healthy reference. The post-exercise profiles revealed these differences more clearly than the at-rest profiles, although their physiological significance remains uncertain. Correspondingly, ΔHR/HR showed no change across the first two visits, followed by a negative change during the final visit.

Overall, the comparisons in Figure 8 and Figure 9 suggest that the 5 min post-exercise harmonic response may provide additional descriptive information about patient-specific CV dynamics beyond that available from at-rest measurements. However, its sensitivity and physiological specificity require further validation.

#### 3.3.3. Normalized APW At-Rest Between the RA and CA

**Figure 10:**
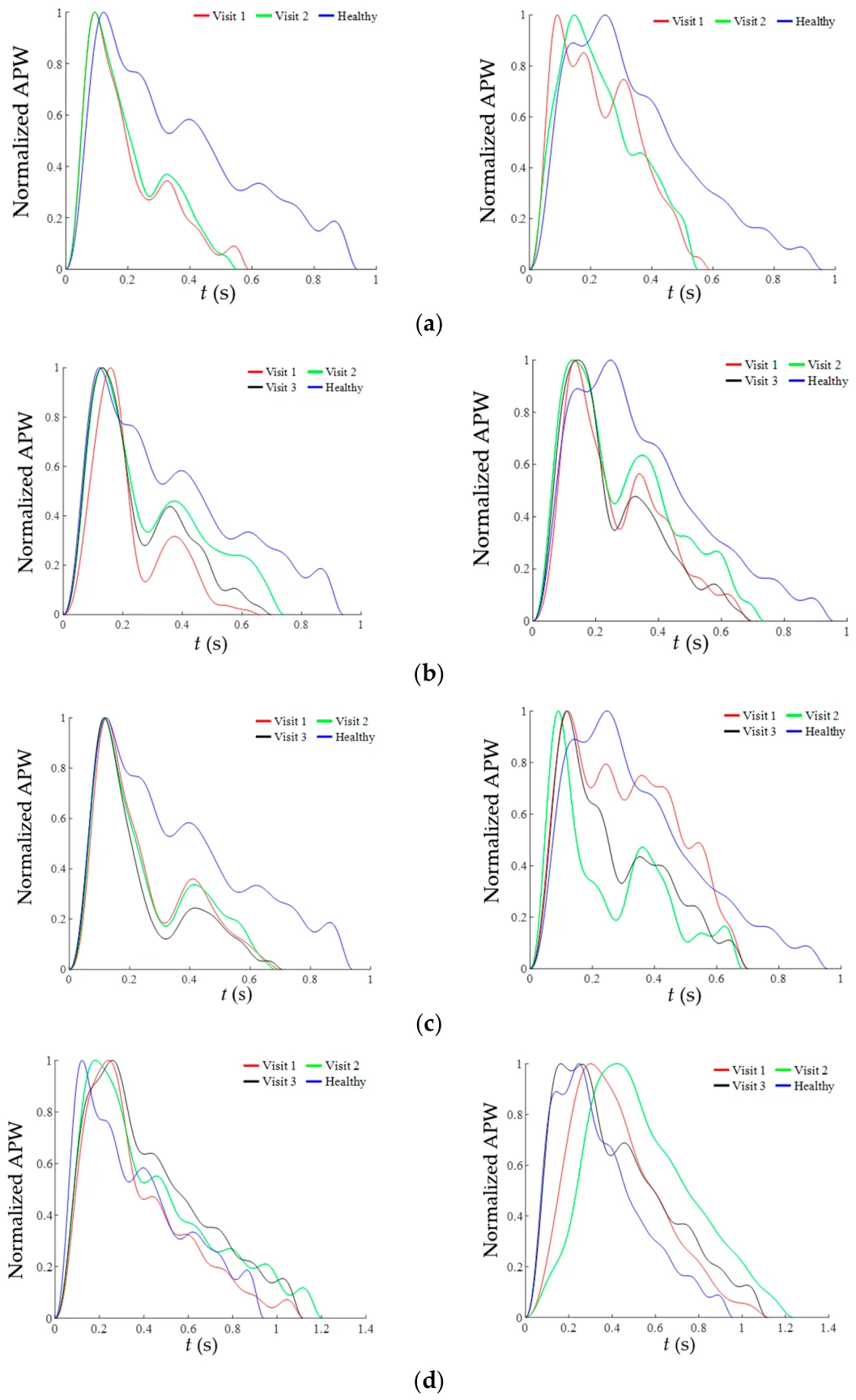

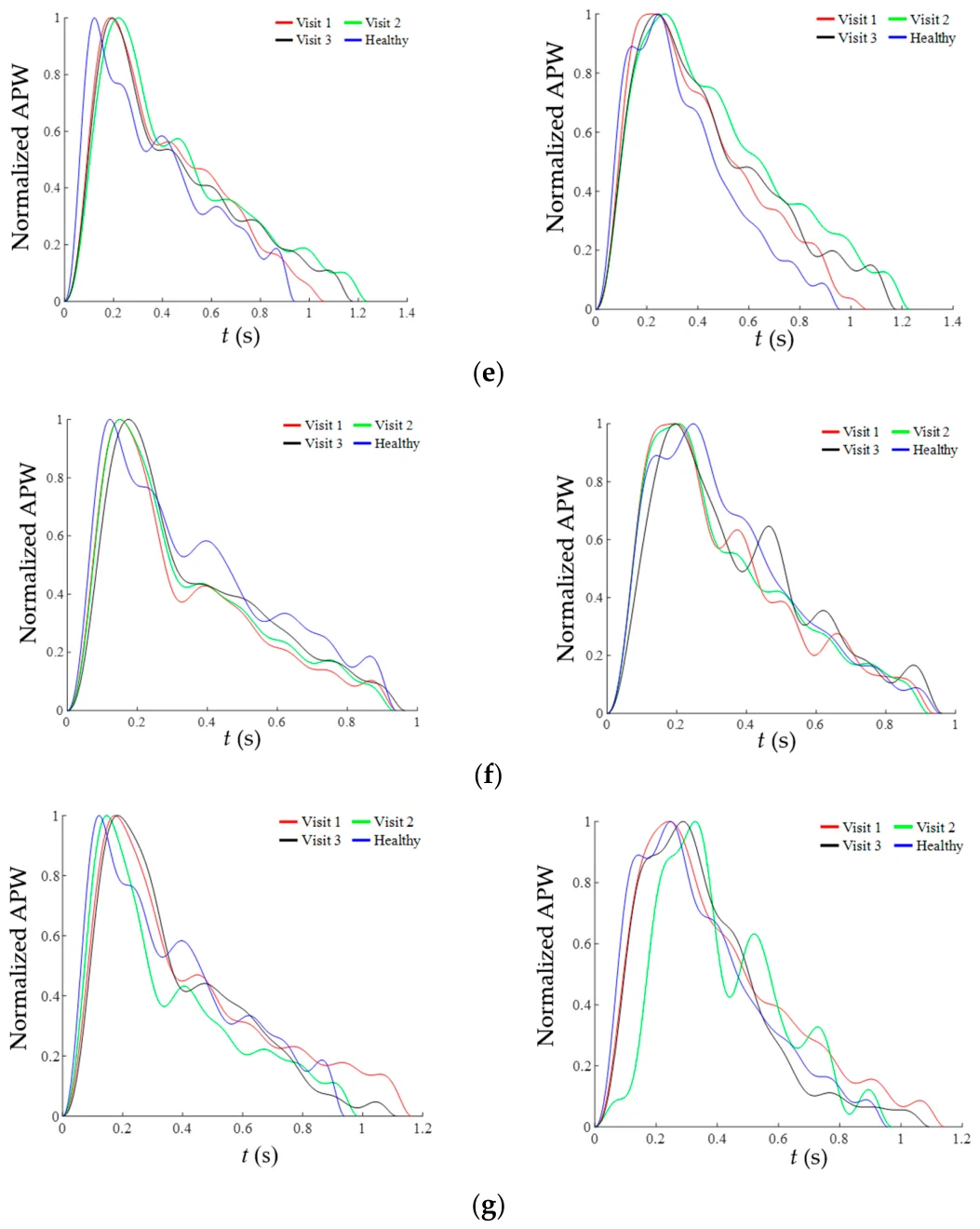
Normalized APW at-rest of the subjects at the RA (left figure) and CA (right figure) (**a**) CR008 (**b**) CR002 (**c**) CR011 (**d**) CR013 (**e**) CR035 (**f**) CR019 (**g**) CR020.

Figure 10 compares the normalized at-rest APWs of the subjects across visits with those of the healthy reference. As discussed in Part I, the DN location in the RA waveform has been proposed as a waveform characteristic potentially associated with arterial stiffness; however, it is not a direct or validated measure of arterial stiffness in the present study. For CR008 (HTx), the DN at the RA was markedly lower than that of the healthy reference, indicating a difference in waveform morphology. Meanwhile, the normalized APWs at the RA remained highly similar across the two visits. In contrast, the normalized APWs at the CA showed substantial inter-visit variation. Given the greater susceptibility of CA-derived APWs to variations in the TCS stack, it remains unclear whether these differences reflect physiological variation, measurement variability, or a combination of both.

The low-DN case subset (CR002 and CR011) exhibited substantially lower DN locations at the RA than the healthy reference. For CR002, the normalized APWs at the CA consistently showed a markedly lower DN than the healthy reference. Although some inter-visit variation was present at both arterial sites, the overall APW patterns remained relatively consistent, suggesting that the effective TCS conditions at the CA may have been comparatively stable for this subject. In contrast, for CR011, the normalized APWs at the RA maintained similar longitudinal patterns, whereas those at the CA varied substantially across visits. Notably, CR011 had a very high BMI (48.8), which may have contributed to the pronounced variability in CA measurements through changes in the TCS stack. Nevertheless, the contribution of TCS-related effects cannot be separated from physiological variation based on the available measurements.

For the low-HR case subset, both CR013 and CR035 exhibited DN locations at the RA comparable to that of the healthy reference. Their normalized APWs at the RA also showed relatively consistent longitudinal patterns. However, the normalized APWs at the CA revealed substantial inter-visit variation for CR013 but only mild variation for CR035. As with the other cases, it remains unclear whether the pronounced CA variation observed for CR013 reflects genuine physiological changes, measurement variability, or a combination of both. It should also be noted that CR013 was diagnosed with non-ST-elevation myocardial infarction (NSTEMI), whereas CR035, who had a cardiac resynchronization therapy defibrillator (CRT-D), was diagnosed with chronic systolic heart failure. Both patients had normal BMI values.

For CR019 and CR020, both subjects exhibited lower DN locations at the RA than the healthy reference and relatively consistent normalized APW patterns across visits at the RA. In contrast, their normalized APWs at the CA showed substantial inter-visit variation. As shown in Supplementary Materials, the CA measurements did not capture the intermittent APW changes as distinctly as the RA measurements, which may have affected the derivation and interpretation of the normalized APWs at the CA.

#### 3.3.4. Intermittent APW Variations in CR019 and CR020

**Figure 11:**
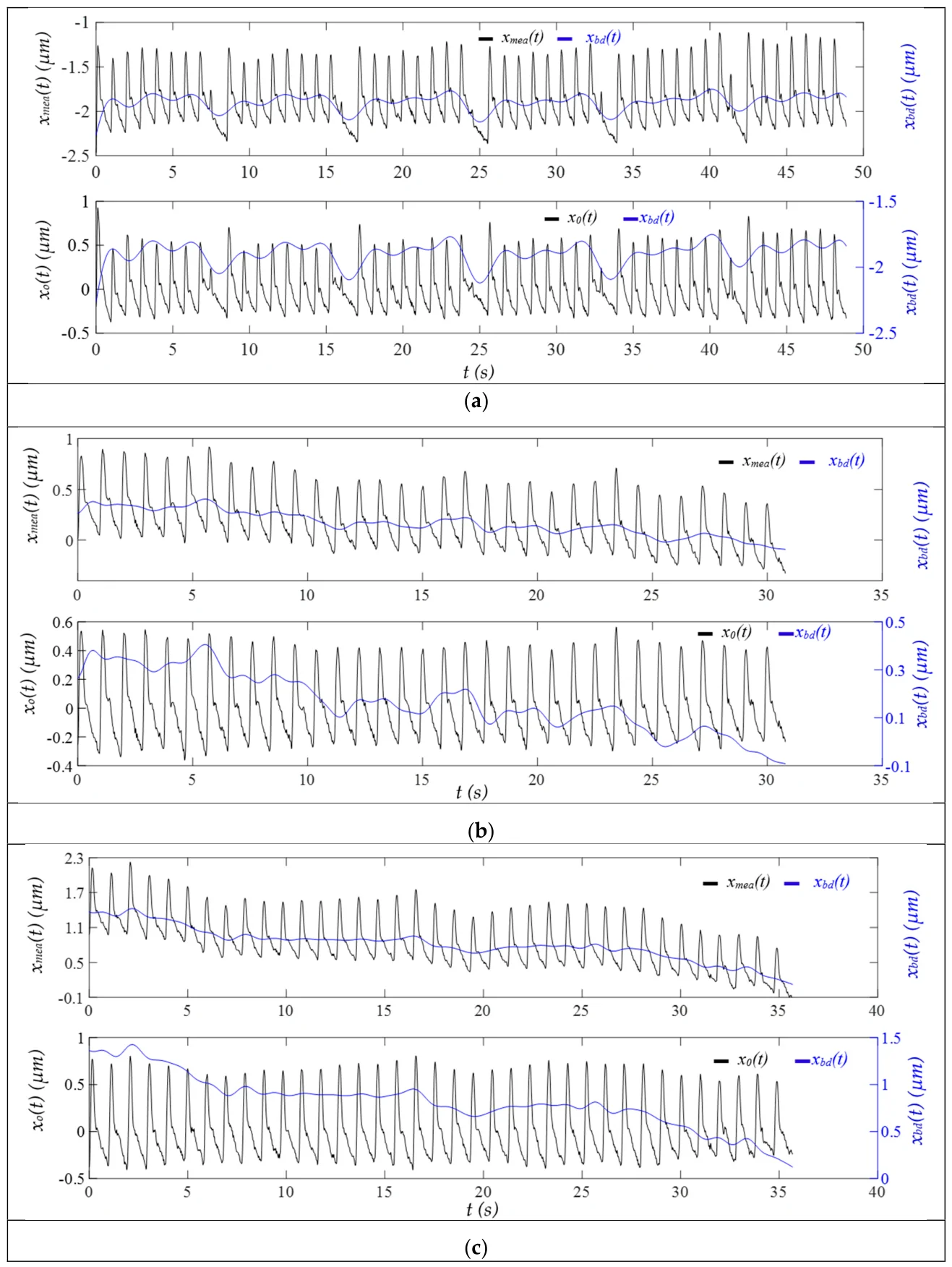
Measured arterial pulse signals at the RA of CR019 at-rest (**a**) Visit 1 (**b**) Visit 2 (**c**) Visit 3. Note that *x*_0_(*t*)* = x_mea_*(*t*) − *x_bd_*(*t*).

**Figure 12:**
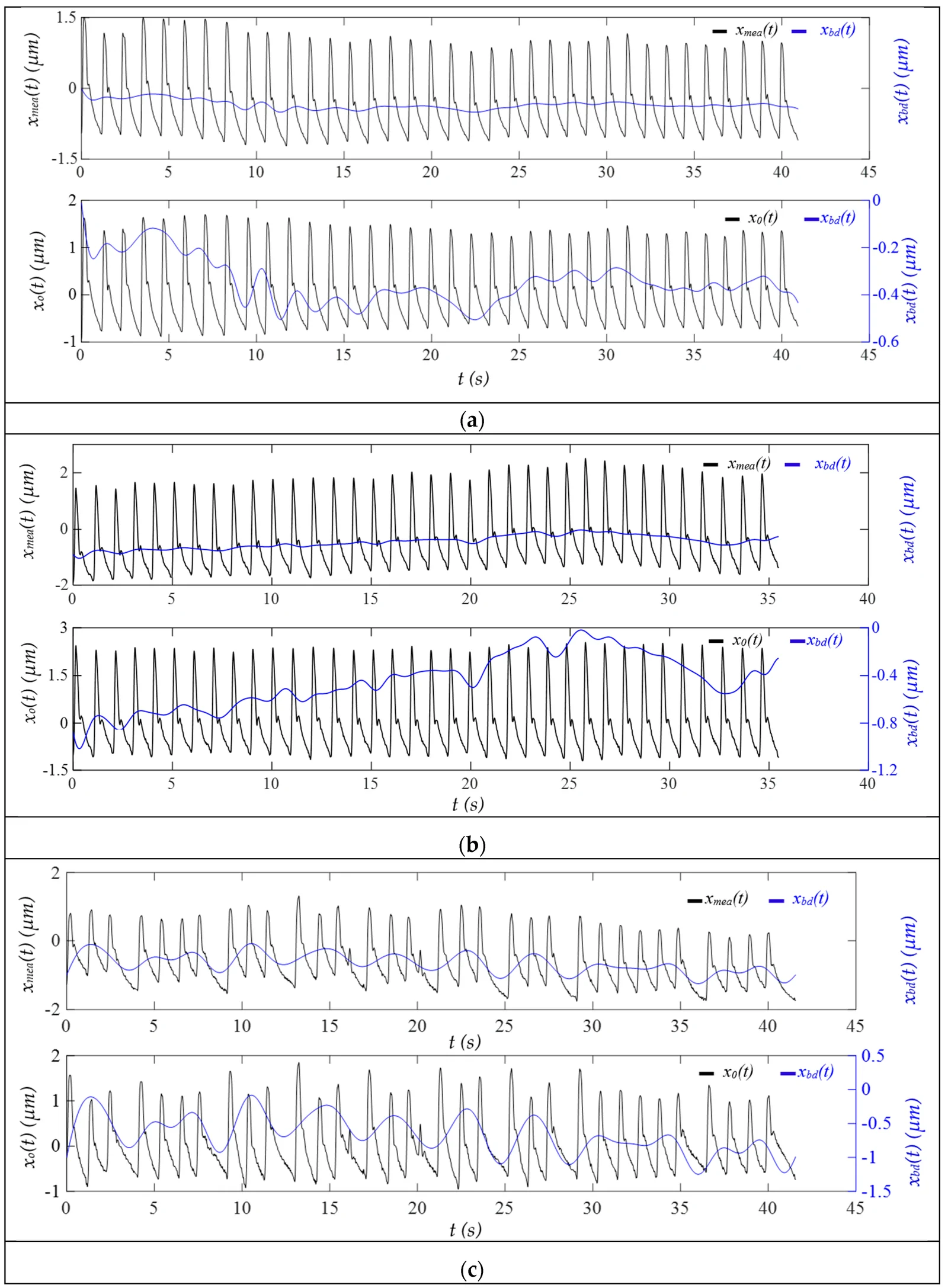
Measured arterial pulse signals at the RA of CR020 at-rest (**a**) Visit 1 (**b**) Visit 2 (**c**) Visit 3. Note that *x*_0_(*t*)* = x_mea_*(*t*) − *x_bd_*(*t*).

Figure 11 and Figure 12 present the measured at-rest pulse signals at the RA for CR019 (AF and MI, 66–70 yr) and CR020 (PCI, 76–80 yr), respectively, where *x*_0_(*t*) denotes the measured pulse signal after removal of the baseline drift *x_bd_*(*t*). Both subjects clearly exhibited intermittent changes occurring every several pulse cycles, revealing transient, repetitive variations in pulse morphology that are largely obscured in the time-averaged APWs shown in Figure 10f,g.

For CR019, the intermittent pattern was most prominent during Visit 1, when the waveform exhibited a rhythmic “swing,” and the APW became distorted and elongated every few beats. During post-exercise recovery, the frequency of these intermittent fluctuations progressively decreased from 5 min to 30 min. Interestingly, this intermittent signature was absent at-rest during Visits 2 and 3, suggesting a more stable pulse morphology during those visits. Similarly, no obvious intermittent signature was observed during post-exercise recovery in these later visits.

In contrast, CR020 demonstrated a progressive increase in intermittency at the RA across the three visits. At Visit 1, no discernible intermittent fluctuations were observed at-rest, but slight intermittent fluctuations appeared at 5 min post-exercise. At visit 2, intermittent fluctuations remained absent at-rest, but pronounced intermittent fluctuations emerged at 5 min post-exercise. By Visit 3, prominent intermittent fluctuations were also present at-rest. However, similar to CR019 at Visit 1, the frequency of intermittent fluctuations progressively decreased during post-exercise recovery from 5 min to 30 min.

Examination of the corresponding CA measurements indicated substantial contamination by MA, particularly respiratory motion during post-exercise recovery. Consequently, the CA measurements did not reveal clear additional features beyond those observed in the RA measurements, whereas the RA measurements provided substantially clearer and more reliable visualization of the intermittent patterns. Additional RA measurements during post-exercise recovery, together with the corresponding CA measurements for CR019 and CR020, are provided in Supplementary Materials.

## 4. Discussion

In this study, the same tactile sensor and SDOF–TF method for arterial pulse measurement and signal processing, respectively, adopted in Part I were applied to a subset of seven patients exhibiting atypical CV characteristics. Although the cohort size was limited, the analysis revealed distinct subject-specific CV patterns that differed markedly from those observed in the general cohort. Both RA and CA measurements were examined to evaluate inter-site differences. Due to the heterogeneous nature of both this subset and the general cohort in Part I, it is difficult to systematically identify all differences between the two groups. Nevertheless, one notable distinction was that the harmonic amplitude responses at 5 and 10 min post-exercise of these seven patients differed distinctly from those observed in the general cohort. Similar to Part I, the effects of exercise were heterogeneous, with no consistent longitudinal trend across subjects or CV parameters. The methodological limitations of this study are identical to those discussed in Part I; therefore, the discussion here focuses primarily on the physiological implications of the analyzed results, in the context of the technical foundation established by the tactile sensor for acquiring arterial pulse measurements with assessed signal quality and the SDOF–TF method for recovering pulse-induced signals from measured sensor data.

### 4.1. Common Features and Individualized CV Signatures

Despite substantial heterogeneity across subjects, several common tendencies were observed. At-rest HR and HRV exhibited relatively limited longitudinal variation for most patients, while higher-order harmonics (5th–7th) remained consistently lower than the healthy reference across visits. In contrast, lower-order harmonics, particularly the 2nd and 3rd harmonics, showed clear inter-subject variability, especially in cases with altered DN morphology.

At the RA, time-based CV indices and waveform-derived features generally provided more consistent subject-specific patterns across visits. In contrast, CA measurements were more susceptible to MA and variations in the TCS stack, which reduced the reliability of CA-derived parameters, particularly during post-exercise recovery. For APW morphology, RA measurements generally provided clearer and more consistent representations of subject-specific patterns, including intermittent APW variations that were not as distinctly captured by CA measurements. These results indicate that RA measurements are more reliable for characterizing individual CV patterns under the current acquisition conditions, whereas CA-derived parameters, particularly those sensitive to signal contamination, must be interpreted cautiously.

Although simultaneous RA–CA measurements provide an opportunity to examine inter-site consistency, agreement between the two sites does not establish longitudinal repeatability or independently validate the physiological accuracy of the extracted CV parameters. In Part I, which focused on subject-specific characterization across 18 patients, detailed CA–RA comparisons were limited to APW morphology in a subset of patients to maintain the study’s focus and scope. In contrast, Part II examines seven selected cases with atypical CV characteristics, allowing more detailed patient-specific comparisons between the two measurement sites. This difference in analytical scope does not imply that CA measurements are as reliable as RA measurements. Compared with their RA-derived counterparts, CA-derived time-based indices may be more susceptible to MA, particularly HRV, RR, and RM, which depend on temporal variations in the instantaneous frequency of a harmonic in the measured pulse signal. Accordingly, CA-derived time-based indices are presented for descriptive comparison only and should not be interpreted as independent evidence of measurement reliability. Longitudinal changes across visits may reflect both physiological variation and measurement-related effects, which cannot be fully distinguished in the absence of independent physiological reference measurements and quantitative repeatability assessments.

### 4.2. Post-Exercise Recovery Better Distinguishes CV Condition than At-Rest Assessment

Across the studied patients, post-exercise measurements revealed more distinct inter-subject differences than at-rest measurements. At-rest harmonic amplitude profiles often showed overlapping patterns, whereas the responses at 5 min post-exercise exhibited more distinct harmonic structures and changes relative to at-rest values. These post-exercise responses also revealed subject-specific dynamic patterns, but varied substantially across patients, with no consistent longitudinal trend across subjects or CV parameters. These findings suggest that post-exercise recovery assessment may provide complementary information for characterizing individual CV patterns beyond that available from at-rest measurements alone. Given the heterogeneous cohort and the absence of independent physiological reference measurements for validating the extracted CV parameters, these differences should be interpreted as descriptive observations rather than direct evidence of impaired CV regulation or disease-specific recovery mechanisms.

### 4.3. APW at the CA Warrants Further Investigation

Normalized APWs at the CA frequently differed from those at the RA, and CA-derived APW morphology exhibited greater inter-visit variability in some subjects. This difference was particularly evident in CR011, whose CA waveforms varied substantially across visits despite relatively consistent patterns at the RA. For CR019 and CR020, the intermittent APW variations observed at the RA were also not captured as distinctly by the CA measurements.

For CR011, whose BMI was 48.8, the greater variability in CA-derived APW morphology may partly reflect measurement-related effects associated with body habitus, including differences in sensor–tissue contact and local tissue mechanics. However, the present data cannot distinguish these potential effects from intrinsic vascular or physiological variation. Therefore, the observed CA–RA differences should not be attributed solely to BMI or interpreted as direct evidence of physiological variation.

The greater variability of CA-derived APWs is likely associated, at least in part, with the higher susceptibility of CA measurements to MA and variations in the TCS stack, particularly during post-exercise recovery. In contrast, RA measurements generally provided clearer and more consistent representations of subject-specific APW morphology across visits. However, these findings are descriptive, and simultaneous physiological reference measurements were not available to independently validate the physiological significance of the observed APW patterns. Thus, the greater clarity and consistency of RA measurements support their relative reliability under the current acquisition conditions but do not, by themselves, establish the physiological validity of the derived features. Further methodological improvements are needed to reduce MA and TCS-stack-related effects and to validate CA- and RA-derived APW features against appropriate physiological reference measurements.

### 4.4. Intermittent Changes in CR019 and CR020

CR019 and CR020 exhibited intermittent APW variations that were not apparent in the other subjects. These variations occurred over multiple pulse cycles and were clearly visible in the measured RA signals but were obscured in the time-averaged APWs.

For CR019, intermittent variations were most prominent during Visit 1 and were not observed at-rest or during post-exercise recovery at visits 2 and 3. In contrast, CR020 exhibited a progressive increase in intermittency across visits, with prominent variations present even at-rest during Visit 3. In both subjects, the frequency of intermittent variations decreased during post-exercise recovery.

The corresponding CA measurements showed substantial contamination by MA, particularly respiratory motion during post-exercise recovery, and did not capture the intermittent APW variations as distinctly as the RA measurements. These findings support the greater relative reliability of RA measurements for characterizing intermittent waveform patterns under the current acquisition conditions.

Several mechanisms may potentially contribute to these intermittent variations, including cardiac rhythm disturbances such as AF, medication effects following PCI, autonomic fluctuations, and respiratory coupling. However, these possibilities cannot be distinguished using the present data, as simultaneous ECG and other physiological reference measurements were unavailable. Therefore, the observed variations are interpreted as descriptive APW patterns rather than evidence of any specific underlying mechanism or confirmed physiological significance.

### 4.5. Complexity and Individual Variability of CV Assessment

The analysis highlights the numerous physiological and measurement-related factors that may influence CV assessment. Even among subjects with the same CV diagnosis, substantial variability was observed, potentially reflecting differences in disease severity, medication effects, autonomic regulation, vascular condition, age, BMI, and individual physiological responses to exercise.

Interpretation of the extracted CV parameters is also complicated by patient-specific factors, including BMI, medication use, comorbidities, and implanted cardiac devices. BMI and local tissue characteristics may affect the TCS stack, particularly at the CA, whereas medications and underlying conditions may influence HR, HRV, and post-exercise recovery. Implanted devices, including CRT-D systems, and heart transplantation may also be associated with altered cardiac rhythm or autonomic regulation. Because these factors were not independently evaluated in this descriptive study, their individual contributions to the observed CV patterns cannot be determined. Future studies should systematically account for these clinical and measurement-related factors when evaluating associations between pulse-derived parameters and CV dynamics.

Furthermore, CV diseases and pharmacological treatments may alter the relationships among CV parameters at rest and during post-exercise recovery, contributing to substantial inter-subject variability that cannot be adequately characterized by a limited number of parameters alone. The simultaneous analysis of approximately 15 CV parameters derived from arterial pulse measurements provides a broader descriptive characterization of individual CV patterns, encompassing time-based indices, harmonic amplitudes, and APW morphology. This approach may support personalized CV assessment and longitudinal monitoring by identifying subject-specific patterns that would be difficult to characterize using individual parameters alone However, given the heterogeneity of the patient cohort and the descriptive nature of this study, the observed patterns cannot be directly attributed to specific CV diagnoses or interpreted as evidence of diagnostic performance. Further studies involving larger, clinically defined cohorts and appropriate physiological reference measurements are needed to determine whether particular CV patterns are consistently associated with specific diagnoses and to establish the validity and potential clinical utility of this approach.

Photoplethysmography (PPG) may provide a complementary measurement channel for future studies combining optical and tactile pulse sensing. Although PPG has a different transduction mechanism and its own sensor–tissue transfer characteristics, its simultaneous acquisition with tactile measurements could help investigate intermittent changes in APW morphology, such as those observed in CR019 and CR020. Concordant changes across two physically distinct sensing modalities and measurement sites would provide additional evidence supporting a physiological origin, although modality-specific artifacts and physiological influences, including cutaneous vasodilation during post-exercise recovery, would still need to be considered. Our previous study [15] demonstrated the application of the SDOF–TF framework to PPG signals for AF detection, supporting further investigation of this approach in multimodal pulse analysis. Future studies could therefore examine synchronized PPG and tactile measurements to assess their complementary capabilities and limitations in characterizing longitudinal APW changes and extracting time-based CV indices.

## 5. Conclusions

This study applied the SDOF–TF framework to arterial pulse measurements acquired using tactile sensors in a subset of cardiac rehabilitation patients exhibiting atypical CV characteristics. The combined analysis of at-rest and post-exercise measurements provided a broader characterization of individual CV patterns than at-rest assessment alone, particularly by revealing recovery-dependent changes in harmonic amplitudes and APW morphology. In selected cases, notably CR019 and CR020, intermittent APW variations were clearly observed in the RA measurements but were less distinctly captured by the corresponding CA measurements.

Substantial inter-subject variability was observed across the approximately 15 CV parameters, reflecting the heterogeneous characteristics of the patient cohort and potentially involving differences in diagnosis, medication status, disease severity, and physiological responses to exercise. RA measurements generally provided clearer and more consistent representations of subject-specific APW patterns across visits, whereas CA measurements exhibited greater variability and susceptibility to MA and TCS-stack-related effects under the current acquisition conditions. These findings support the descriptive value of simultaneous analysis of multiple CV parameters for characterizing individual CV patterns and their changes during post-exercise recovery. However, the present study does not establish that specific CV patterns are associated with particular CV diagnoses, nor does it independently validate the physiological significance of all extracted parameters. Further studies involving larger, clinically defined cohorts, appropriate physiological reference measurements, and improved acquisition protocols are needed to establish the validity and clinical utility of this approach for longitudinal CV monitoring and potential personalized assessment, with particular attention to improving the reliability of CA measurements.

## Figures and Tables

**Table 1:** Baseline demographic and clinical characteristics of the 18 cardiac patients and the healthy reference, including CV conditions, cardiac rhythms, beta-blocker use, and implanted devices.

	Subject ID	Gender	Age	BMI	Reason for CR	Rhythm	Beta Blocker	Devices
	Healthy	Male	41	19.9				
HTx	CR-008	Male	56–60	20.8	HTx	SR	No	None
Low HR	CR-002	Male	46–50	33.7	PCI	SR	Yes	None
	CR-011	Male	51–55	48.8	NSTEMI and PCI	SR	Yes	None
Low DN location	CR-013	Male	61–65	24.2	NSTEMI	SR	No	None
	CR-035	Male	66–70	28.3	HF	SR	Yes	CRT-D
Intermittent APW variations	CR-019	Male	66–70	27	MI	AF	Yes	None
	CR-020	Male	76–80	24.2	PCI	SR	Yes	None

**Table 2:** At-rest HR and maximum HR during exercise in the 7 cardiac patients and the healthy reference, along with the corresponding absolute and normalized maximum HR differences (note that HR_max_ is missing for CR003 at Visit 3).

Subject	Visit	HR			HR_max_ (bpm)	ΔHR_max_ − HR_sensor_ (bpm)	ΔHR_max_/HR (bpm)
		HR_medical_	HR_sensor_ (CA)	HR_sensor_ (RA)			
Healthy			62.82	63.72	106.75	43.03	68%
**CR008**	Visit 1	101.33	101.83	101.81	112.00	10.19	10%
	Visit 2	108.00	108.95	108.92	128.00	19.08	18%
CR002	Visit 1	90.00	86.82	91.22	119.00	27.78	30%
	Visit 2	81.00	81.56	80.87	122.00	41.13	51%
	Visit 3	86.00	85.81	85.85			
CR011	Visit 1	83.00	86.52	86.46	114.00	27.54	32%
	Visit 2	88.33	88.27	87.96	127.00	39.04	44%
	Visit 3	82.33	85.37	84.74	131.00	46.26	55%
**CR013**	Visit 1	53.00	53.56	53.85	72.00	18.15	34%
	Visit 2	49.00	48.52	49.57	71.00	21.43	43%
	Visit 3	56.00	53.94	53.86	101.00	47.14	**88%**
CR035	Visit 1	55.67	56.46	56.68	63.00	6.32	11%
	Visit 2	47.67	48.36	48.36	62.00	13.64	28%
	Visit 3	51.33	50.64	50.58	58.00	7.42	15%
CR019	Visit 1	61.33	59.98	61.62	77.00	15.38	25%
	Visit 2	63.00	64.96	64.38	84.00	19.62	30%
	Visit 3	62.00	62.37	62.30	78.00	15.70	25%
CR020	Visit 1	51.67	52.16	51.76	79.00	27.24	53%
	Visit 2	60.00	62.33	60.94	86.00	25.06	41%
	Visit 3	52.33	50.43	50.65	109.00	58.35	**115%**

## Data Availability

Due to institutional policy and applicable privacy regulations, individual-level patient data will not be shared with external investigators or repositories. These restrictions are necessary to protect patient confidentiality and comply with institutional governance of clinical data resources.

## Supplementary Materials

The following supporting information can be downloaded at: https://www.mdpi.com/article/10.3390/diagnostics16213451/s1.

## Abbreviations

The following abbreviations are used in this manuscript:

CRT-D	Biventricular Implantable Cardioverter Defibrillator
HF	Heart Failure
MI	Myocardial Infarction
MVR	Mitral valve replacement
NSTEMI	Non-ST-segment Elevation myocardial Infarction
PCI	Percutaneous Coronary Intervention
SR	Sinus Rhythm
AF	Atrial Fibrillation
CR	Cardiac rehabilitation
CV	Cardiovascular
APW	Arterial pulse waveform
TCS	Tissue-contact-sensor
TVSP	Time-varying system parameters
MA	Motion artifacts
DN	Dicrotic notch
SDOF–TF	Single-degree-of-freedom time-frequency
BD	Baseline drift
HR	Heart rate
HRV	Heart rate variability
RSA	Respiratory sinus arrhythmia
RM	Respiration modulation
RR	Respiration rate
CA	Carotid artery
RA	Radial artery
PF	Physiological factors other than respiration
HTx	Heart transplant

## References

[1] Rahman M.M., Hasan M., May J.F., Herre J.M., Reynolds L.J., Hao Z. (2026). Subject-Specific Cardiovascular Signatures Revealed by Arterial Pulse Signals in Patients with Heart Disease Part I: General Cases. Preprints.

[2] Keeley E.C., Toth Z.K., Goldberg A.D. (2000). Long-term assessment of heart rate variability in cardiac transplant recipients. J. Heart Lung Transplant..

[3] Cornelissen V.A., Vanhaecke J., Aubert A.E., Fagard R.H. (2012). Heart rate variability after heart transplantation: A 10-year longitudinal follow-up study. J. Cardiol..

[4] Wdowczyk J., Makowiec D., Dorniak K., Gruchała M. (2016). Visualization of Heart Rate Variability of Long-Term Heart Transplant Patient by Transition Networks: A Case Report. Front. Physiol..

[5] Fernandes-Silva M.M., Almeida F.D.M., D’Avila V.M., Gellatti M.P., Guimarães G.V. (2025). Cardiac rehabilitation in patients with heart failure: Clinical recommendation based on a review of the evidence. Heart Fail. Rev..

[6] Vanderlaan R.D., Conway J., Manlhiot C., McCrindle B.W., Dipchand A.I. (2012). Enhanced exercise performance and survival associated with evidence of autonomic reinnervation in pediatric heart transplant recipients. Am. J. Transplant..

[7] de Lima J.B., Soares D.D.S., Ferrari F., Carvas Junior N., Carvalho G., Tobar Leitão S.A., Goldraich L.A., Clausell N., Stein R. (2020). Exercise training modalities for heart transplant recipients: A systematic review and network meta-analysis protocol. BMJ Open.

[8] Sidhu S., Marine J.E. (2020). Evaluating and managing bradycardia. Trends Cardiovasc. Med..

[9] Kusumoto F.M., Schoenfeld M.H., Barrett C., Edgerton J.R., Ellenbogen K.A., Gold M.R., Goldschlager N.F., Hamilton R.M., Joglar J.A., Kim R.J. (2019). 2018 ACC/AHA/HRS Guideline on the Evaluation and Management of Patients With Bradycardia and Cardiac Conduction Delay: A Report of the American College of Cardiology/American Heart Association Task Force on Clinical Practice Guidelines and the Heart Rhythm Society. Circulation.

[10] Hamza M.M., Noreddine B., Abdeslem B.Z., Benyssaad Y., Ilham B.S. (2024). Classifying Abnormal Arterial Pulse Patterns in Cardiovascular Diseases: A Photoplethysmography and Machine Learning Approach. Trait. Signal.

[11] Mulder M.P., Broomé M., Donker D.W., Westerhof B.E. (2022). Distinct morphologies of arterial waveforms reveal preload-, contractility-, and afterload-deficient hemodynamic instability: An in silico simulation study. Physiol. Rep..

[12] Koester C., Ibrahim A.M., Cancel M., Labedi M.R. (2020). The Ubiquitous Premature Ventricular Complex. Cureus.

[13] Sattar Y., Hashmi M.F. (2026). Premature Ventricular Complex. StatPearls.

[14] Choi A., Shin H. (2018). Quantitative Analysis of the Effect of an Ectopic Beat on the Heart Rate Variability in the Resting Condition. Front. Physiol..

[15] Rahman M.M., Hasan M., May J., Herre J., Reynolds L., Hao Z. (2026). Radial and carotid arterial pulse signals for assessing cardiovascular function at rest and during post-exercise recovery in a heart transplant patient: A case study. Med. Sens. Imaging.

[16] Cole Christopher R., Blackstone Eugene H., Pashkow Fredric J., Snader Claire E., Lauer Michael S. (1999). Heart-Rate Recovery Immediately after Exercise as a Predictor of Mortality. N. Engl. J. Med..

[17] Kaikkonen P., Rusko H., Martinmäki K. (2008). Post-exercise heart rate variability of endurance athletes after different high-intensity exercise interventions. Scand. J. Med. Sci. Sports.

[18] Parekh A., Lee C.M. (2005). Heart rate variability after isocaloric exercise bouts of different intensities. Med. Sci. Sports Exerc..

