# Supplementary material for: Subject-Specific Cardiovascular Signatures Revealed by Arterial Pulse Signals in Patients with Heart Diseases—Part II: Special Cases
Source: Diagnostics (Basel). 2026 Oct 22;16(21):3451. doi: 10.3390/diagnostics16213451 (PMC13762237; doi:10.3390/diagnostics16213451)
Supplement: Supplementary file 1 [file diagnostics-16-03451-s001.zip › diagnostics-4558591-supplementary.pdf]

Supplementary Document for
Subject-Specific Cardiovascular Signatures Revealed by Arterial Pulse Signals in Patients With Heart Diseases — Part II: General Cases

Supplementary A

Measured arterial pulse signals at the RA and the CA of CR019 at-rest and during post-exercise recovery

Note: The corresponding CA measurements may have been chosen from a different time segment from the RA measurements to obtain higher-quality pulse signals

1st Visit

RA measurement

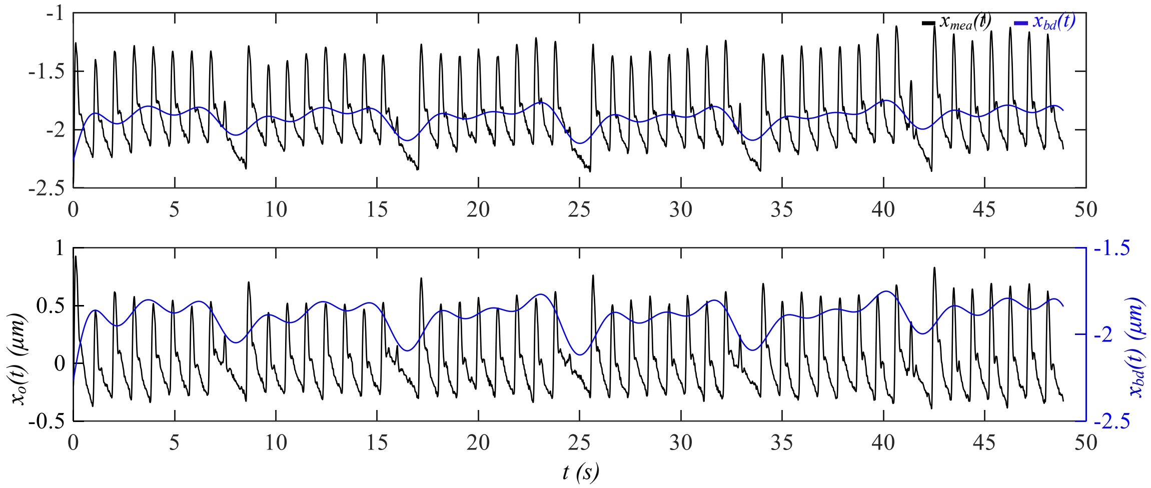

Figure S1. At-rest

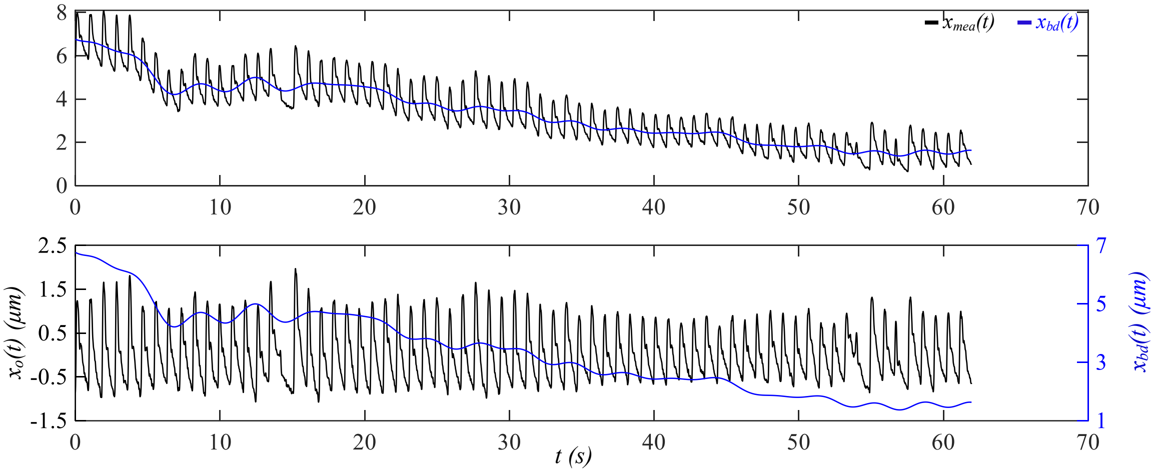

Figure S2. 5min post-exercise

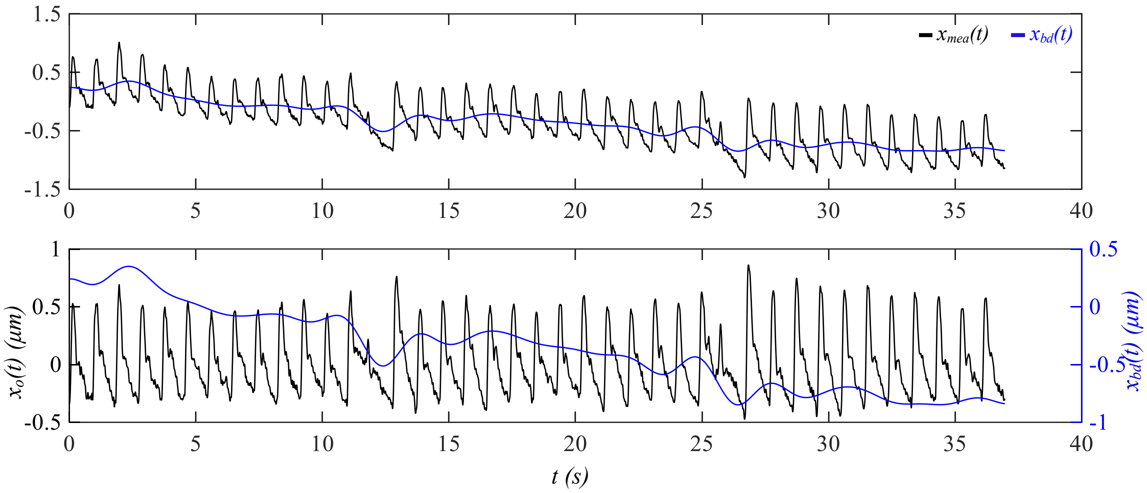

Figure S3. 10min post-exercise

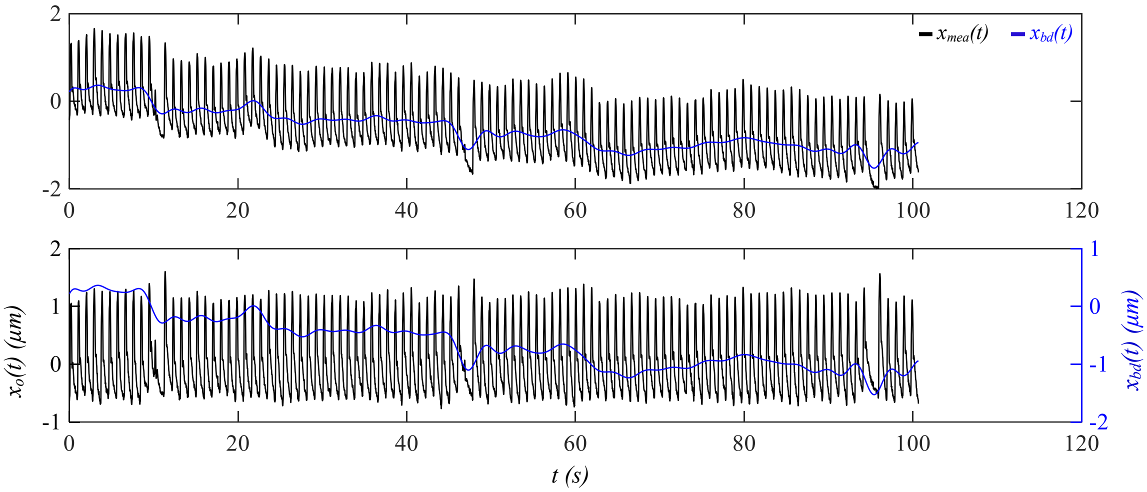

Figure S4. 20min post-exercise

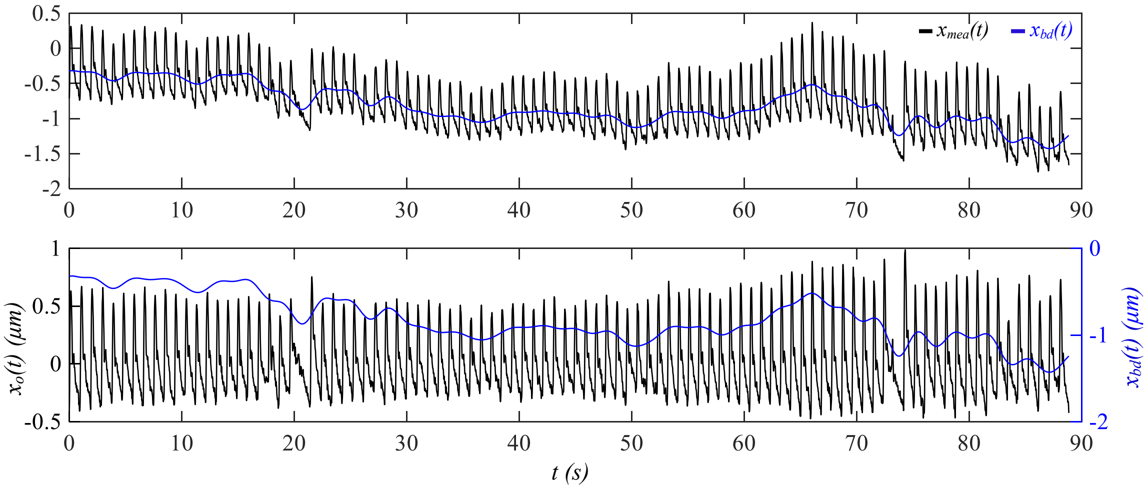

Figure S5. 30min post-exercise

CA measurements

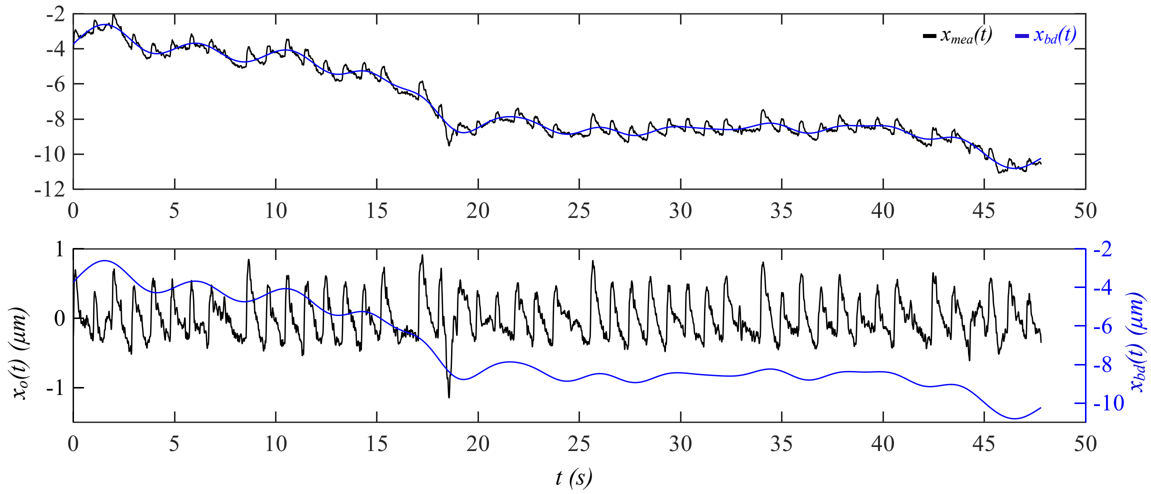

Figure S6. At-rest

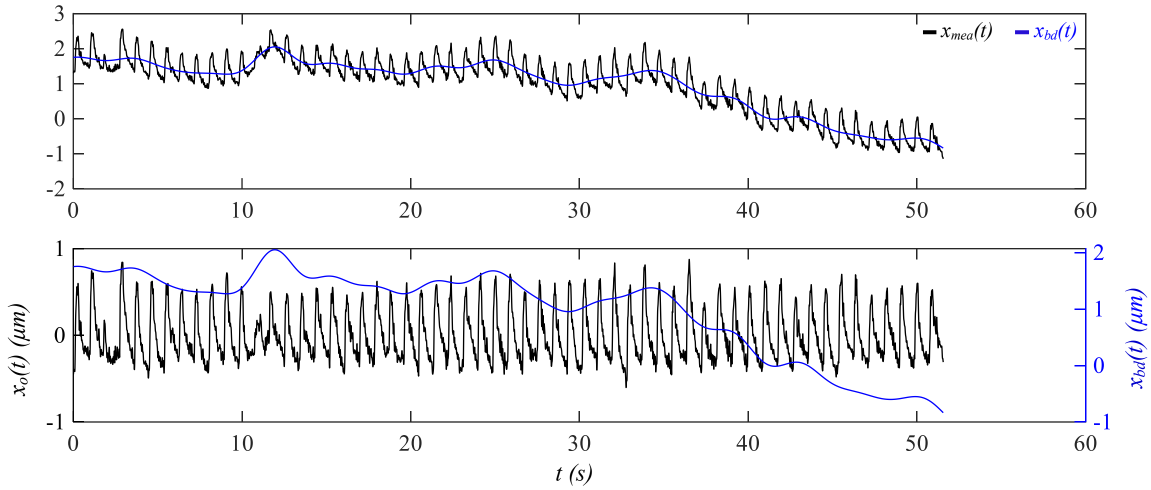

Figure S7. 5min post-exercise (different time segment from RA)

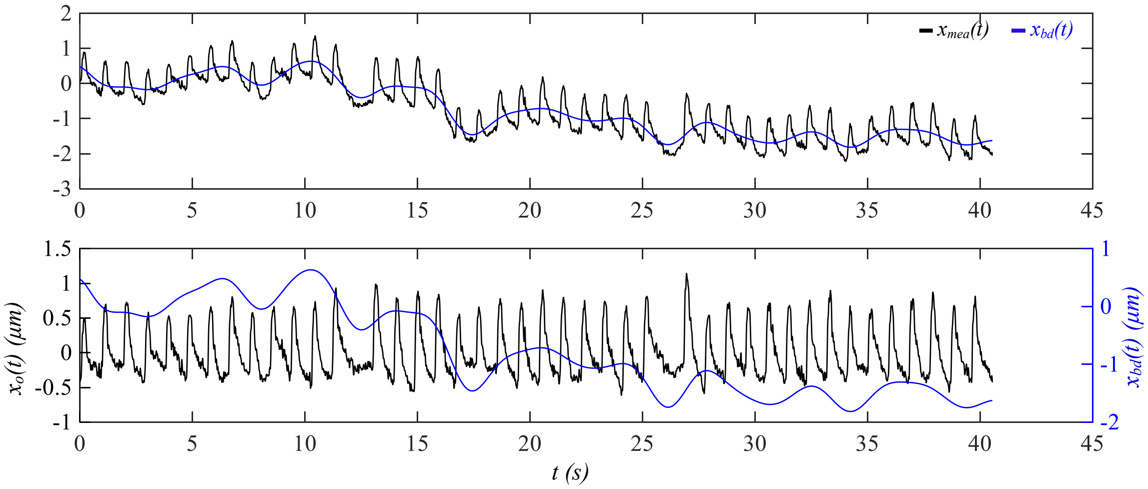

Figure S8. 10min post-exercise (different time segment from RA)

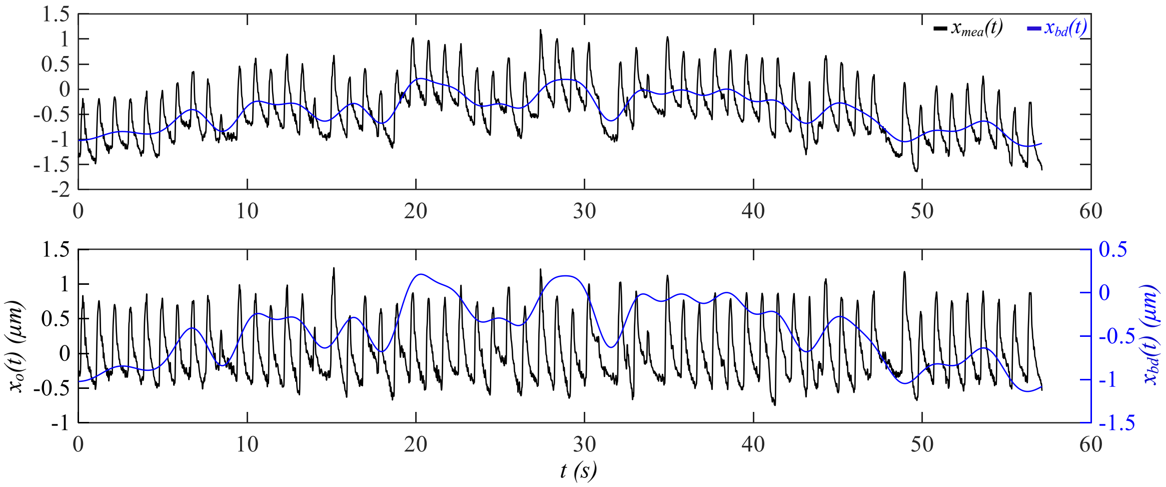

Figure S9. 20min post-exercise (different time segment from RA)

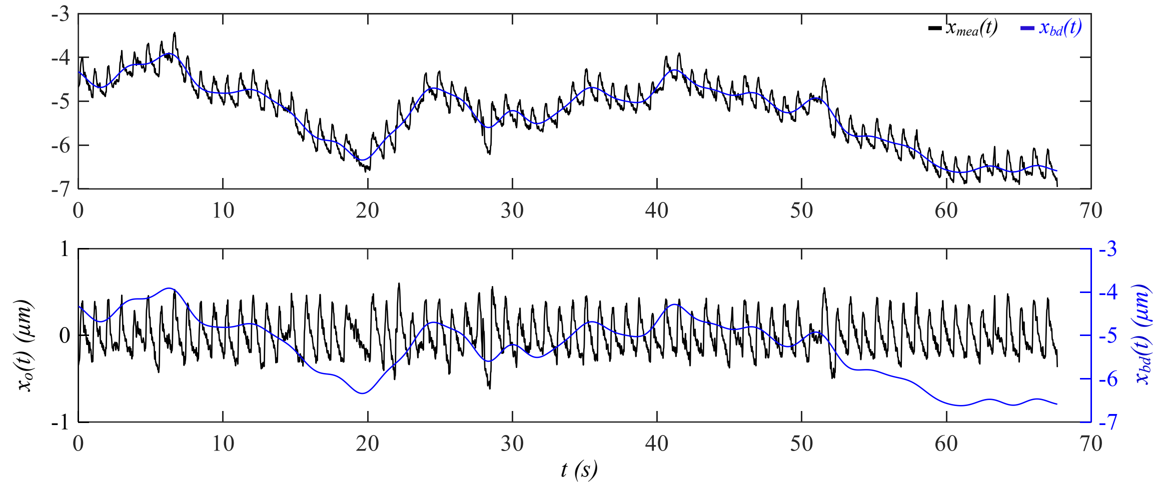

Figure S10. 30min post-exercise (different time segment from RA)

2nd Visit

RA measurements

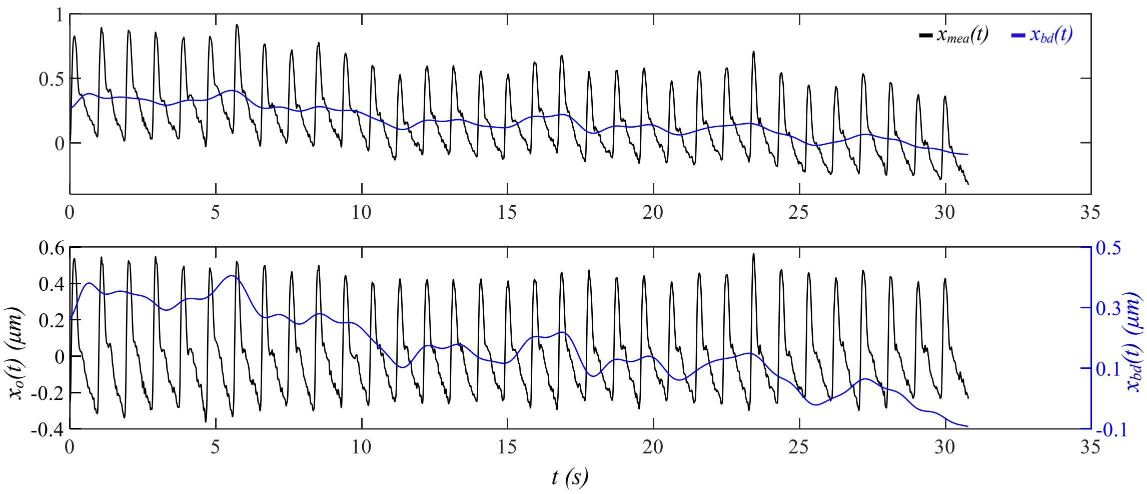

Figure S11. At-rest

CA measurements

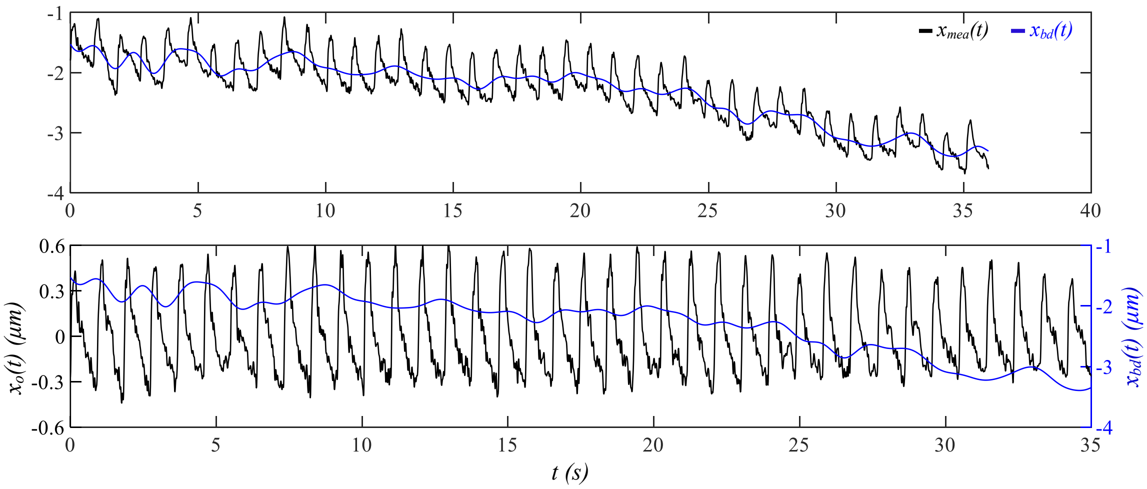

Figure S12. At-rest (different time segment from RA)

3rd Visit

RA measurements

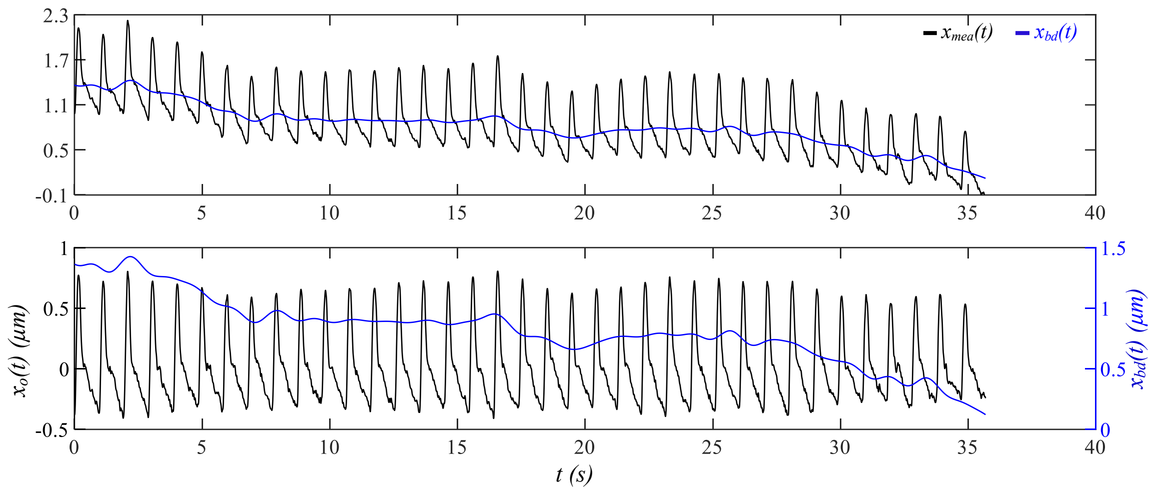

Figure S13. At-rest

CA measurements

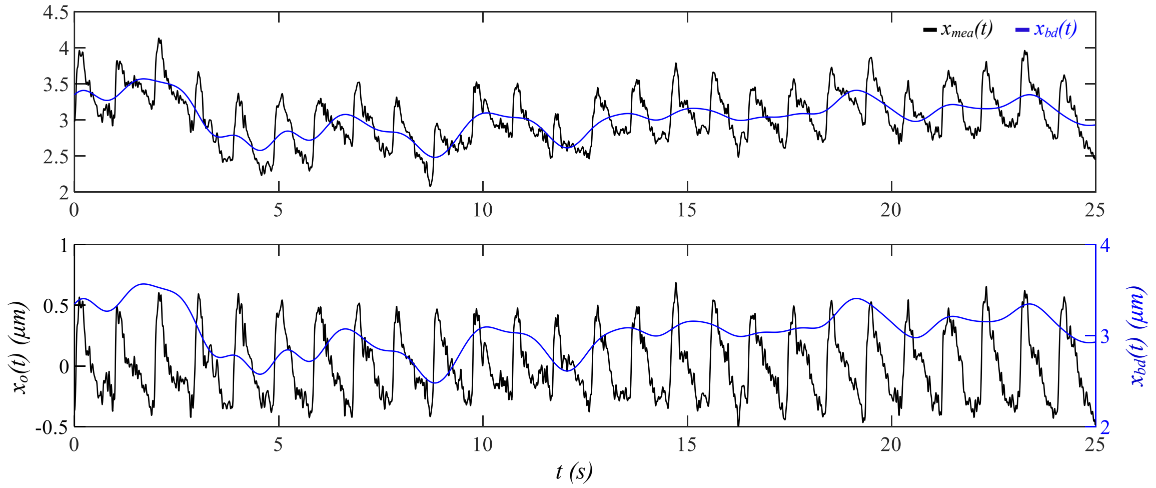

Figure S14. At-rest

Supplementary B

Measured arterial pulse signals at the RA and the CA of CR020 at-rest and during post-exercise recovery

Note: The corresponding CA measurements may have been chosen from a different time segment from the RA measurements to obtain higher-quality pulse signals

1st Visit

RA measurement

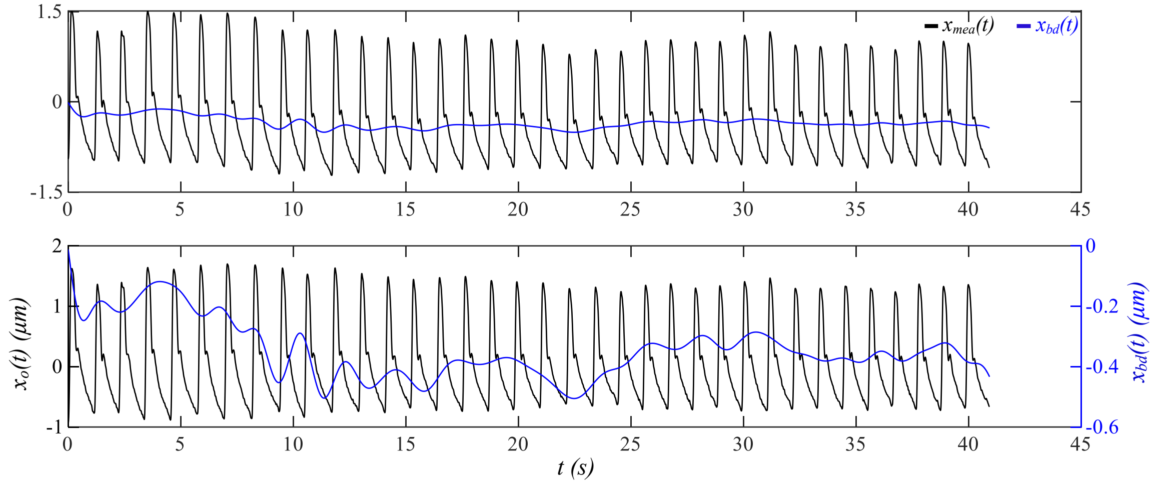

Figure S15. At-rest

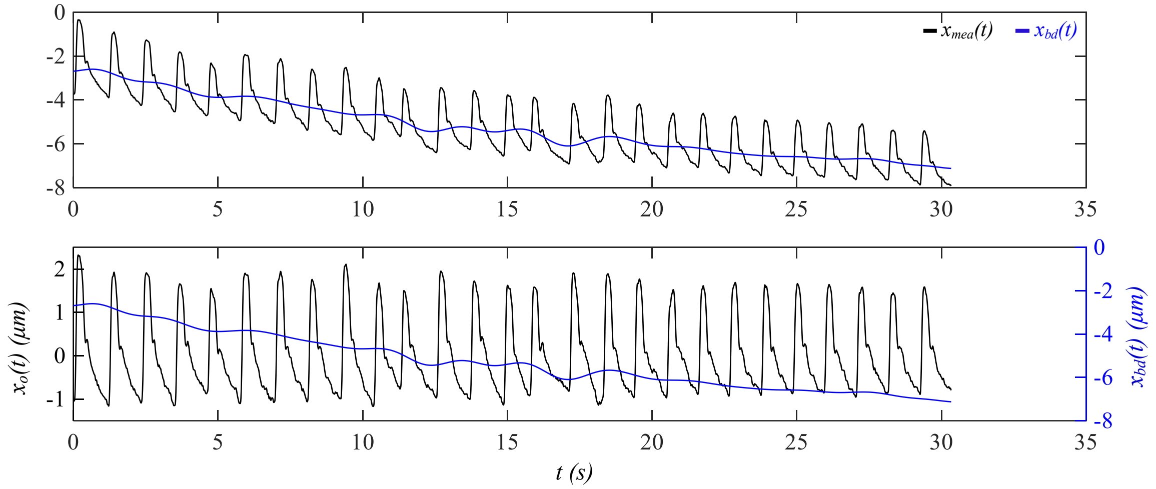

Figure S16. 5min post-exercise

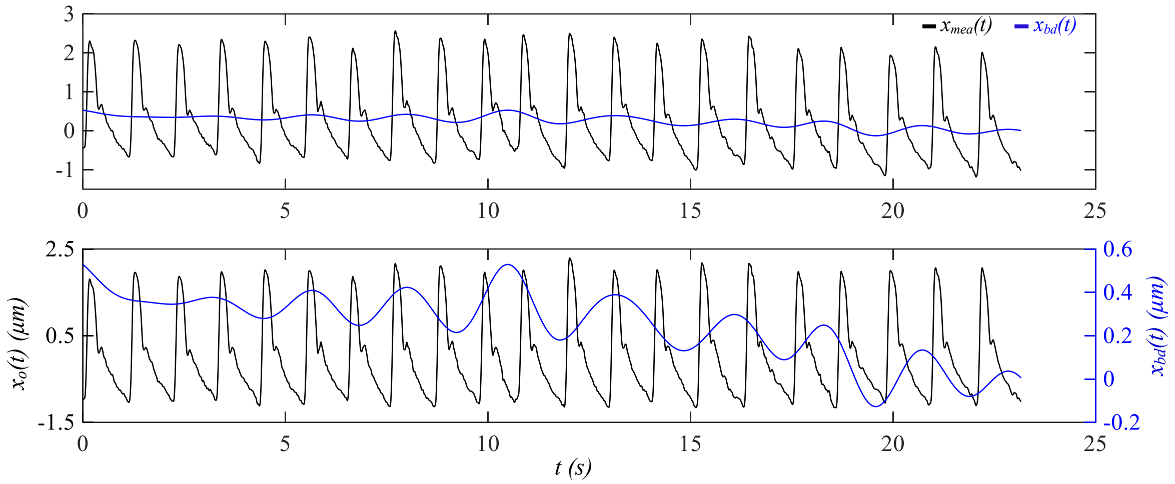

Figure S17. 10min post-exercise

CA measurement

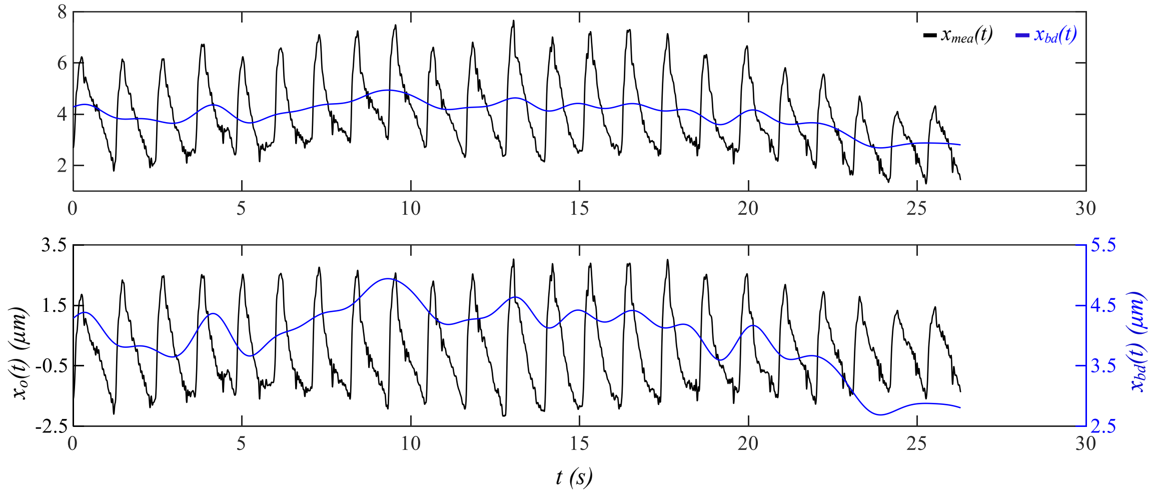

Figure S18. At-rest (different time segment from RA)

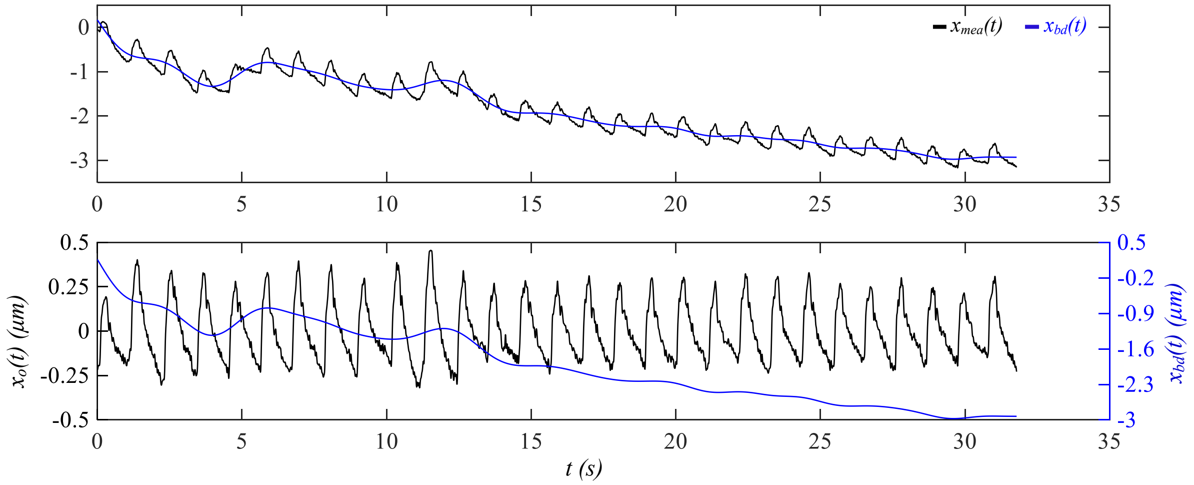

Figure S19. 5min post-exercise (different time segment from RA)

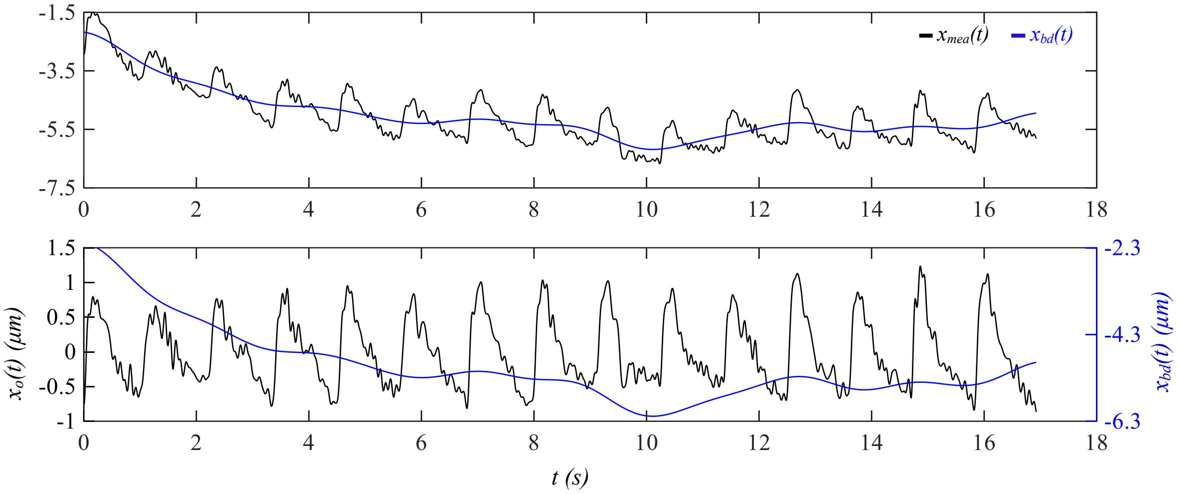

Figure S20. 10min post-exercise (different time segment from RA)

2nd Visit

RA measurement

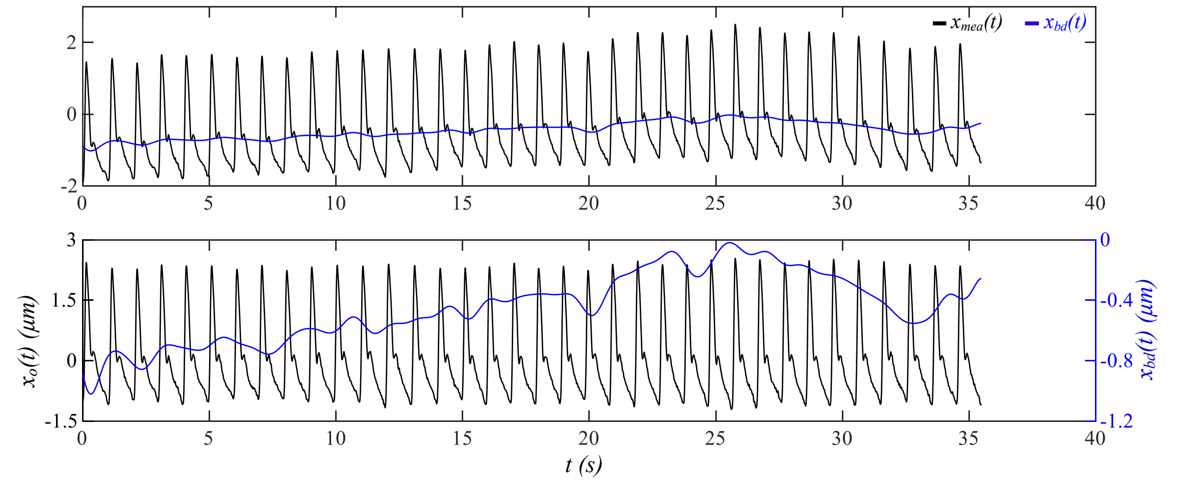

Figure S21. At-rest

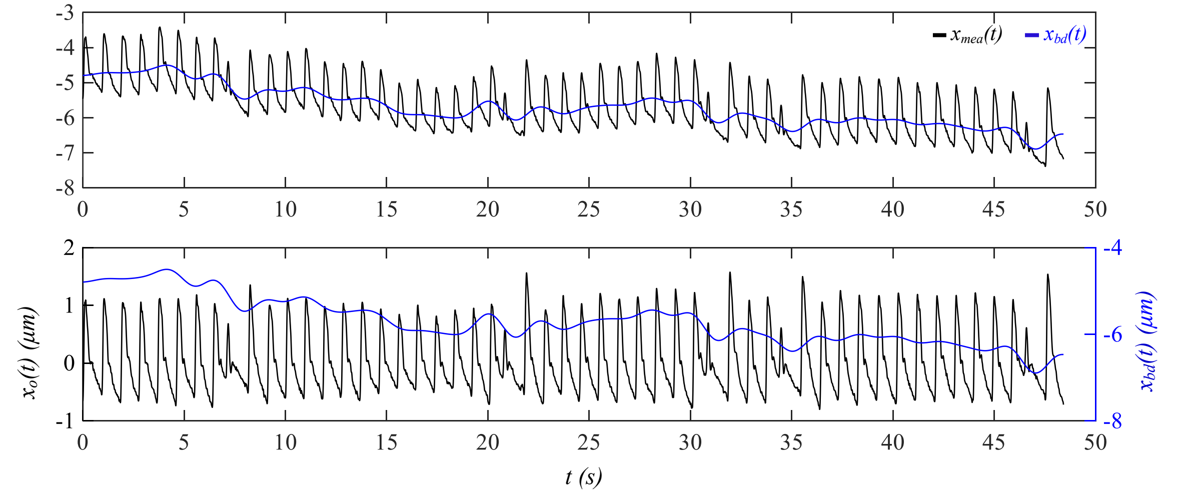

Figure S22. 5min post-exercise

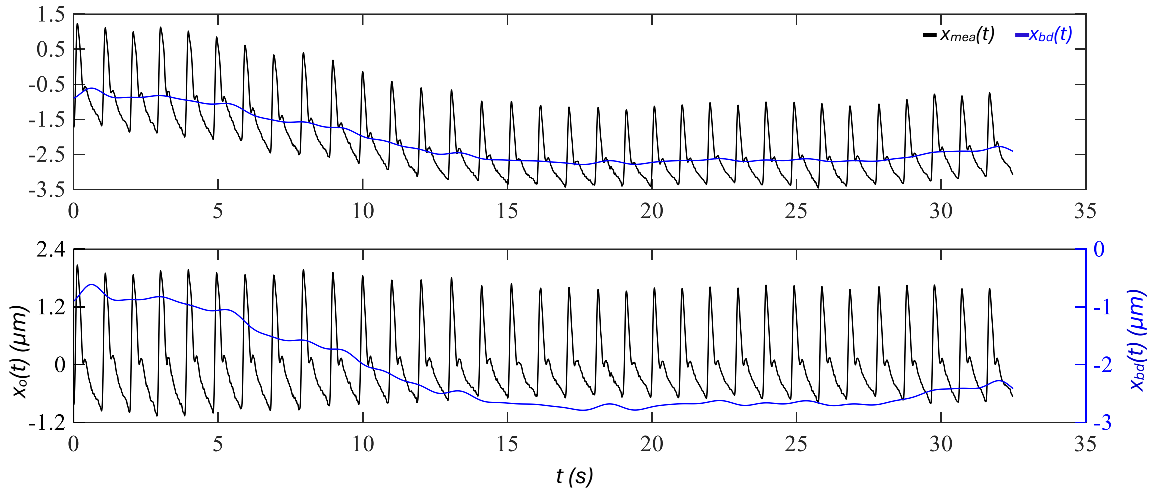

Figure S23. 10min post-exercise

CA measurement

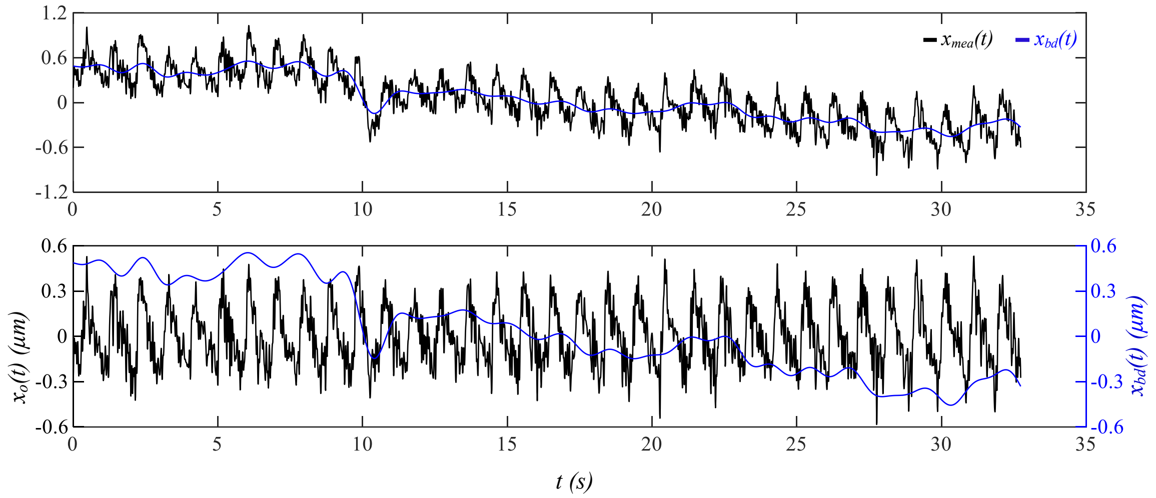

Figure S24. At-rest (different time segment from RA)

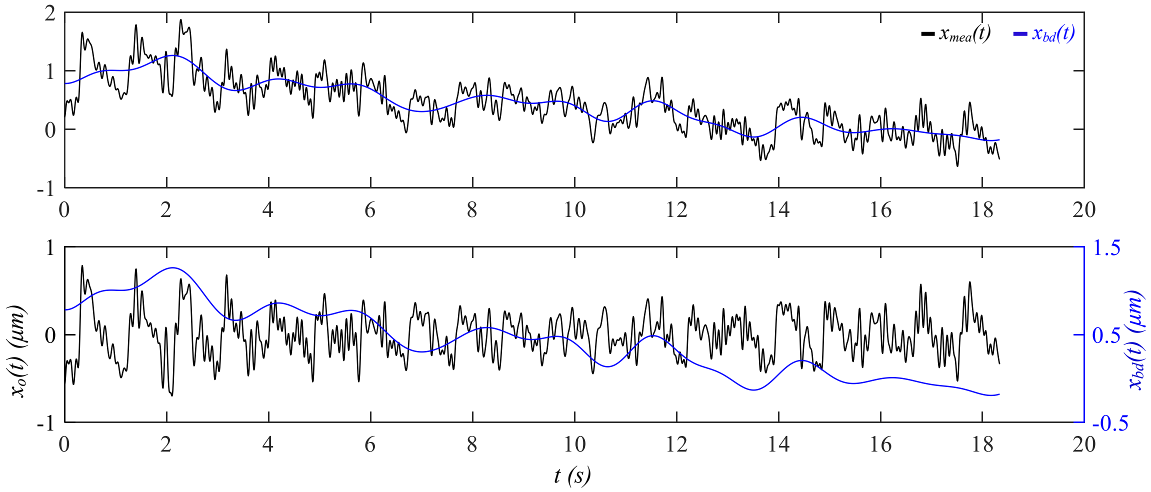

Figure S25. 5min post-exercise (too much noise)

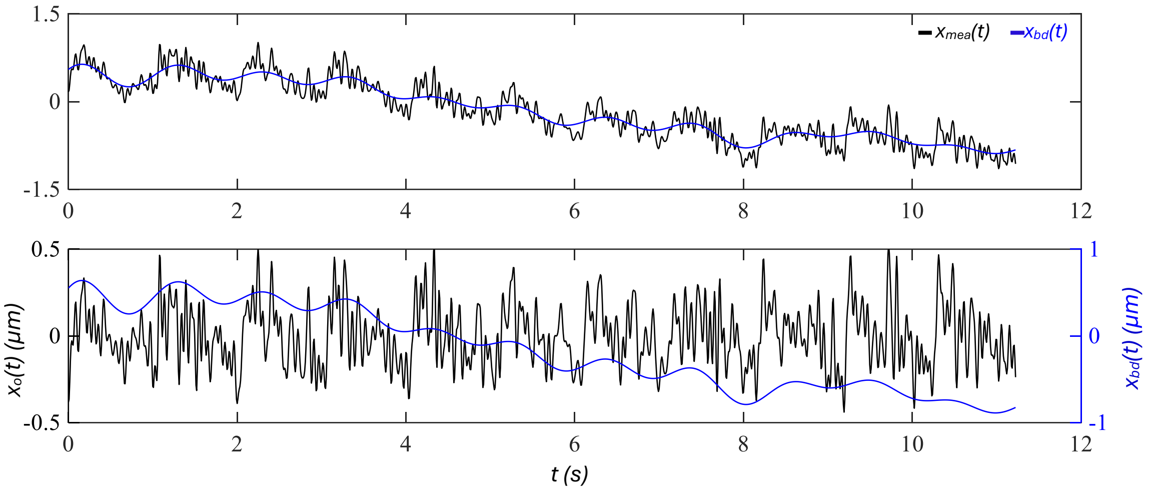

Figure S26. 10min post-exercise (too much noisy)

3rd Visit

RA measurement

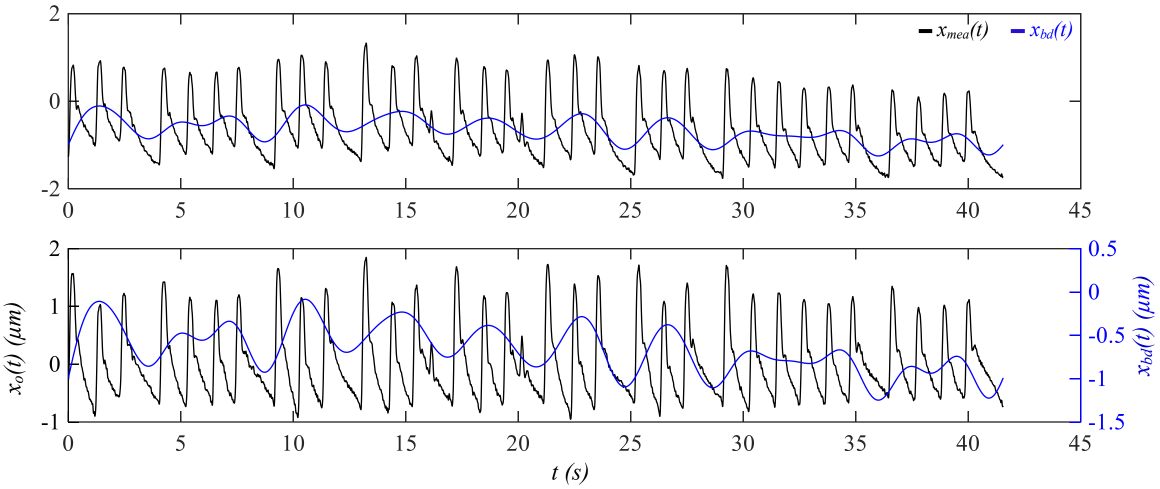

Figure S27. At-rest

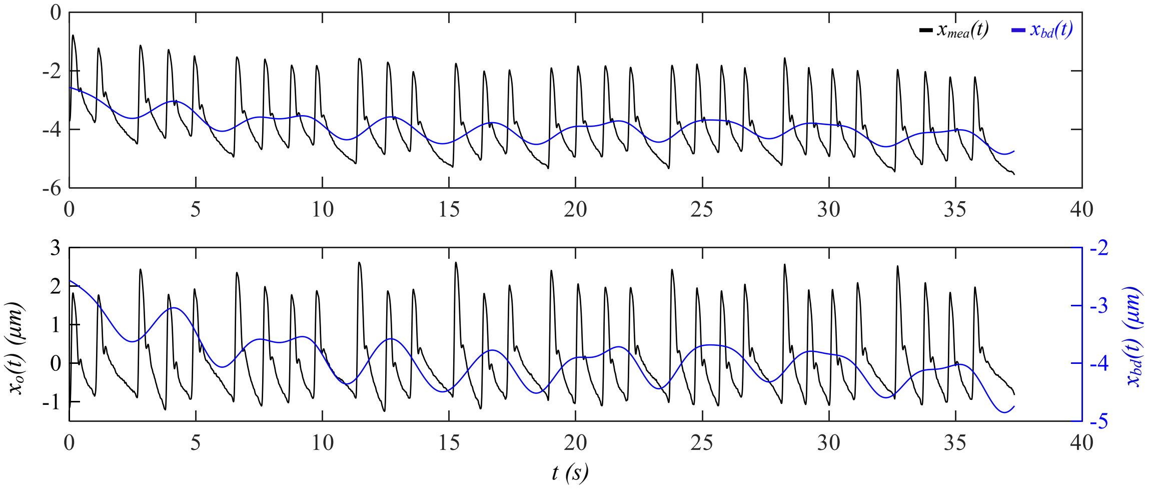

Figure S28. 10min post-exercise

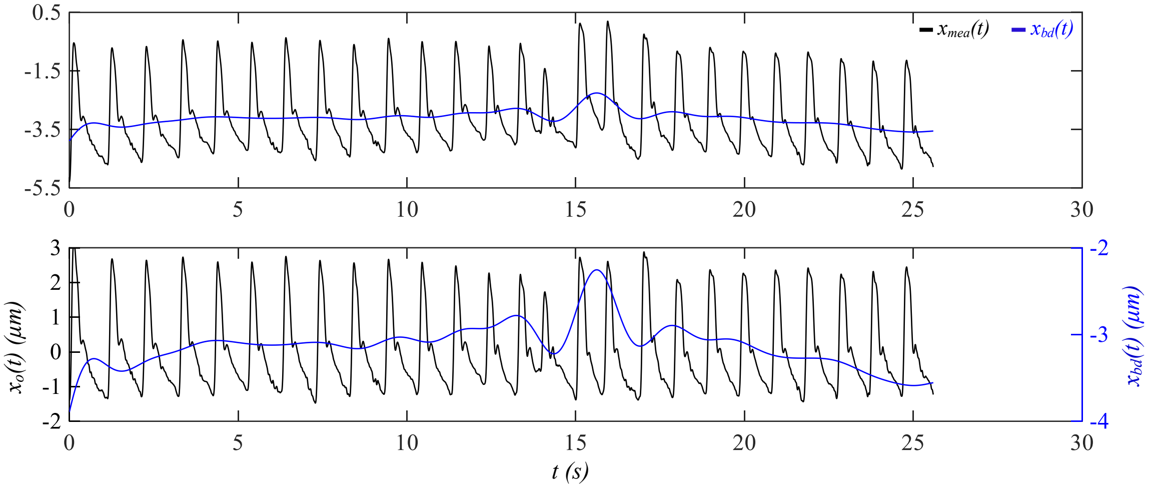

Figure S29. 20min post-exercise

CA measurement

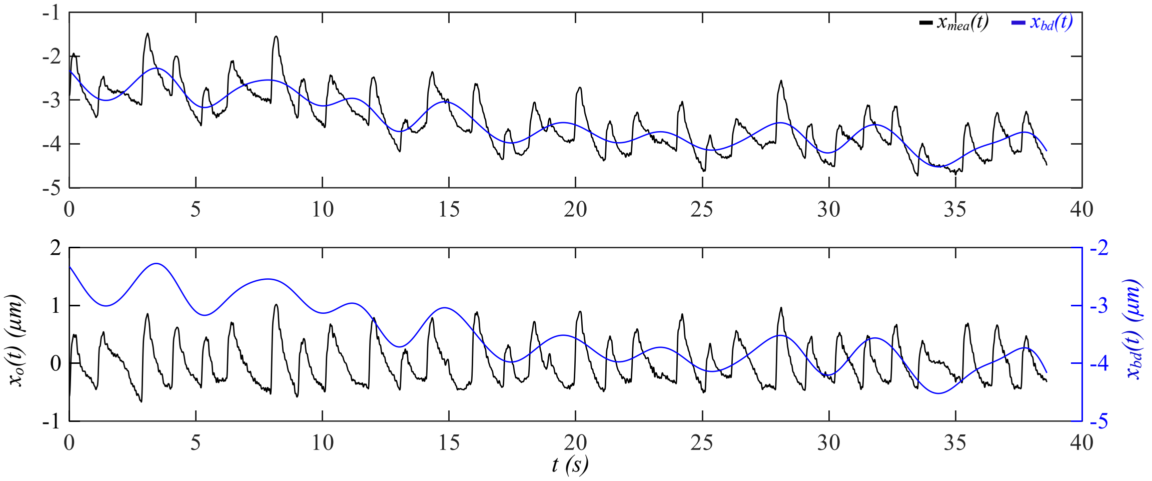

Figure S30. At-rest

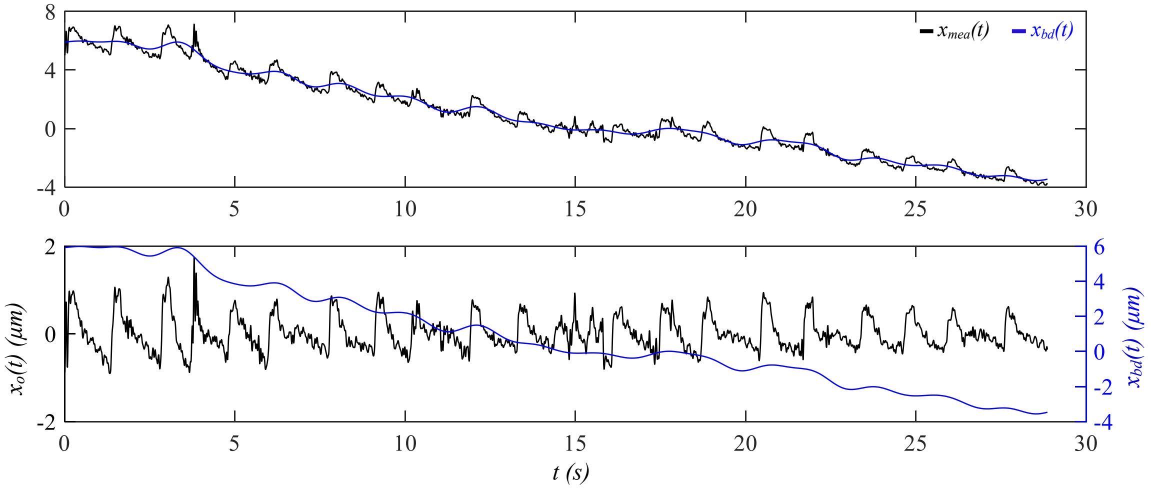

Figure S31. 10min post-exercise (different time segment from RA)

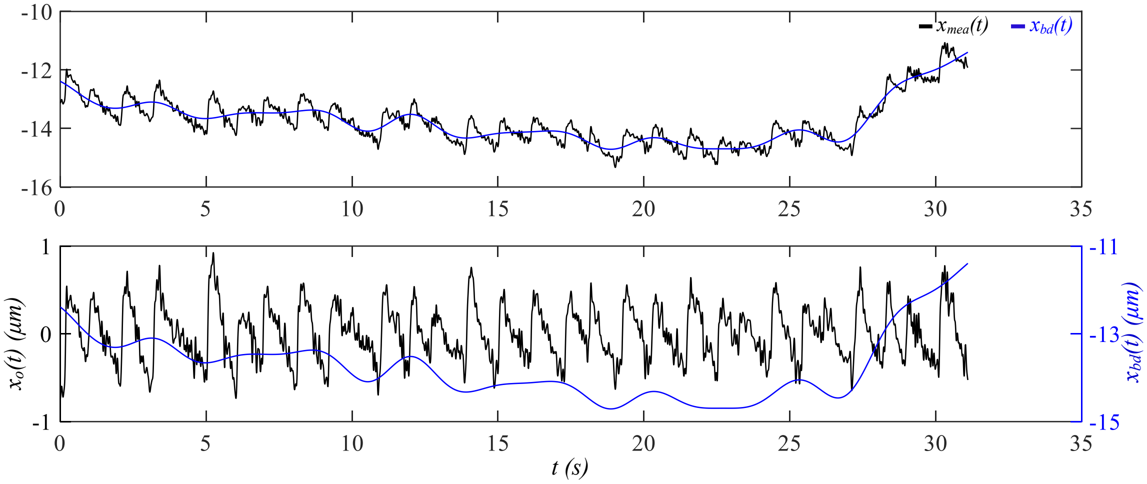

Figure S32. 20min post-exercise
